# Supplementary material for: Incidence, risk factors, and control of Rabies in Ethiopia: A systematic review and meta-analysis
Source: PLoS Negl Trop Dis. 2025 Mar 19;19(3):e0012874. doi: 10.1371/journal.pntd.0012874 (PMC11922250; doi:10.1371/journal.pntd.0012874)
Supplement: S1 Information — (DOCX) [file pntd.0012874.s001.docx]

**Supplementary Information-I**

1. **Search terms**

| **Concept-1** | **Concept-2** | **Concept-3** |
| --- | --- | --- |
| "incidence"[MeSH Terms] "epidemiology"[Subheading**]** | **-** | **-** |
| **Incidence**  Epidemiology  Prevalence  Burden  Exposure  Outbreak  Epidemic  Endemic  Morbidity  **“Risk factors”**  **Mortality**  Death  Fatality  **Control**  ”Control strategies”  ”Control methods”  Prevention  “Prevention strategies”  “Prevention methods”  “Prevention and control”  “Infection control”  “Post-Exposure Prophylaxis”  “Pre-exposure Prophylaxis”  Treatment | Rabies  “Rabies virus”  “Rabies virus infection”  “Animal rabies”  “Dog rabies”  “Rabid dog”  “Rabid animal”  “Human rabies”  “Domestic animal rabies”  “Wild animal rabies”  “Wildlife rabies” | Ethiopia  “Addis Ababa”  “Dire Dawa”  “Benishangul-Gumuz”  Afar  Oromia  Somali  Tigray  Gambella  Harari  “Southern nations, nationalities and peoples’ region”  SNNPR  Amhara  “Sidama region” |

1. **Search strings**

| **Database** | **Search string** |
| --- | --- |
| **PubMed** | ("Incidence"[MeSH Terms] OR "Epidemiology"[MeSH Subheading] OR "Incidence"[Title/Abstract] OR "Epidemiology"[Title/Abstract] OR "Prevalence"[Title/Abstract] OR "Burden"[Title/Abstract] OR "Exposure"[Title/Abstract] OR "Outbreak"[Title/Abstract] OR "Epidemic"[Title/Abstract] OR "Endemic"[Title/Abstract] OR "Morbidity"[Title/Abstract] OR "Risk factors"[Title/Abstract] OR "Mortality"[Title/Abstract] OR "Death"[Title/Abstract] OR "Fatality"[Title/Abstract] OR "Control"[Title/Abstract] OR "Control strategies"[Title/Abstract] OR "Control methods"[Title/Abstract] OR "Prevention"[Title/Abstract] OR "Prevention strategies"[Title/Abstract] OR "Prevention methods"[Title/Abstract] OR "Prevention and control"[Title/Abstract] OR "Infection control"[Title/Abstract] OR "Post-Exposure Prophylaxis"[Title/Abstract] OR "Pre-exposure Prophylaxis"[Title/Abstract] OR "Treatment"[Title/Abstract]) AND "english"[Language] AND (("Rabies"[Title/Abstract] OR "Rabies virus"[Title/Abstract] OR "Rabies virus infection"[Title/Abstract] OR "Animal rabies"[Title/Abstract] OR "Dog rabies"[Title/Abstract] OR "Rabid dog"[Title/Abstract] OR "Rabid animal"[Title/Abstract] OR "Human rabies"[Title/Abstract] OR "Domestic animal rabies"[Title/Abstract] OR "Wild animal rabies"[Title/Abstract] OR "Wildlife rabies"[Title/Abstract]) AND "english"[Language]) AND (("Ethiopia"[Title/Abstract] OR "Addis Ababa"[Title/Abstract] OR "Dire Dawa"[Title/Abstract] OR "Benishangul-Gumuz"[Title/Abstract] OR "Afar"[Title/Abstract] OR "Oromia"[Title/Abstract] OR "Somali"[Title/Abstract] OR "Tigray"[Title/Abstract] OR "Gambella"[Title/Abstract] OR "Harari"[Title/Abstract] OR "southern nations nationalities and peoples region"[Title/Abstract] OR "SNNPR"[Title/Abstract] OR "Amhara"[Title/Abstract] OR "Sidama region"[Title/Abstract]) AND "english"[Language]) |
| **Scopus** | ( TITLE-ABS-KEY ( incidence OR epidemiology OR prevalence OR burden OR exposure OR outbreak OR epidemic OR endemic OR morbidity OR "Risk factors" OR mortality OR death OR fatality OR control OR "Control strategies" OR "Control methods" OR prevention OR "Prevention strategies" OR "Prevention methods" OR "Prevention and control" OR "Infection control" OR "Post-Exposure Prophylaxis" OR "Pre-exposure Prophylaxis" OR treatment ) ) AND ( TITLE-ABS-KEY ( rabies OR "Rabies virus" OR "Rabies virus infection" OR "Animal rabies" OR "Dog rabies" OR "Rabid dog" OR "Rabid animal" OR "Human rabies" OR "Domestic animal rabies" OR "Wild animal rabies" OR "Wildlife rabies" ) ) AND ( TITLE-ABS-KEY ( ethiopia ) ) |
| **Embase** | ('incidence'/exp OR incidence OR 'epidemiology'/exp OR epidemiology OR 'prevalence'/exp OR prevalence OR 'burden'/exp OR burden OR 'exposure'/exp OR exposure OR 'outbreak'/exp OR outbreak OR 'epidemic'/exp OR epidemic OR endemic OR 'morbidity'/exp OR morbidity OR 'risk factors'/exp OR 'risk factors' OR 'mortality'/exp OR mortality OR 'death'/exp OR death OR 'fatality'/exp OR fatality OR 'control'/exp OR control OR 'control strategies' OR 'control methods' OR 'prevention'/exp OR prevention OR 'prevention strategies' OR 'prevention methods' OR 'prevention and control'/exp OR 'prevention and control' OR 'infection control'/exp OR 'infection control' OR 'post-exposure prophylaxis'/exp OR 'post-exposure prophylaxis' OR 'pre-exposure prophylaxis'/exp OR 'pre-exposure prophylaxis' OR 'treatment'/exp OR treatment) AND ('rabies'/exp OR rabies OR 'rabies virus'/exp OR 'rabies virus' OR 'rabies virus infection'/exp OR 'rabies virus infection' OR 'animal rabies' OR 'dog rabies' OR 'rabid dog' OR 'rabid animal' OR 'human rabies'/exp OR 'human rabies' OR 'domestic animal rabies' OR 'wild animal rabies' OR 'wildlife rabies') AND ('ethiopia'/exp OR ethiopia) |
| **Web of science** | ((incidence OR epidemiology OR prevalence OR burden OR exposure OR outbreak OR epidemic OR endemic OR morbidity OR "Risk factors" OR mortality OR death OR fatality OR control OR "Control strategies" OR "Control methods" OR prevention OR "Prevention strategies" OR "Prevention methods" OR "Prevention and control" OR "Infection control" OR "Post-Exposure Prophylaxis" OR "Pre-exposure Prophylaxis" OR treatment)) AND ALL=(rabies OR "Rabies virus" OR "Rabies virus infection" OR "Animal rabies" OR "Dog rabies" OR "Rabid dog" OR "Rabid animal" OR "Human rabies" OR "Domestic animal rabies" OR "Wild animal rabies" OR "Wildlife rabies")) AND ALL=(Ethiopia)) |
