# Supplementary material for: Incidence, risk factors, and control of Rabies in Ethiopia: A systematic review and meta-analysis
Source: PLoS Negl Trop Dis. 2025 Mar 19;19(3):e0012874. doi: 10.1371/journal.pntd.0012874 (PMC11922250; doi:10.1371/journal.pntd.0012874)
Supplement: S3 Information — (PDF) [file pntd.0012874.s003.pdf]

Supplementary Information - III

Incidence rate of suspected rabies in cattle

Study name	Rate	Lower limit	Upper limit
Alemu et al., 2022 (1)	0.009111617	-0.003516208	0.021739443
Jemberu et al., 2013 (2)	0.000198869	0.000068944	0.000328794
Jibat et al., 2016 (3)	0.018801867	0.015741560	0.021862175
Menghistu et al., 2018 (4)	0.000002996	0.000001301	0.000004691
Wakgari et al., 2022 (5)	0.000175000	0.000155915	0.000194085
	0.000180574	0.000009642	0.000351505

Statistics for each study; Rate and 95% CI

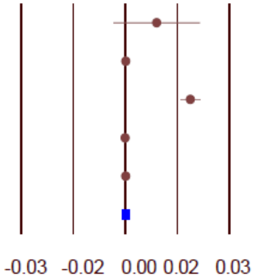

Test of null: Z-value=2.071, P=0.000
Heterogeneity: Q-value (df) =465.133 (4), P= 0.000
I-squared = 99.140
Tau-squared = 0.000

a. Forest plot for incidence rate of rabies in cattle

Incidence rate of suspected rabies in dogs

Study name	Rate	Lower limit	Upper limit
Jemberu et al., 2013 (2)	0.004128295	0.001884169	0.006372420
Menghistu et al., 2018 (4)	0.000152823	0.000058104	0.000247543
Wakgari et al., 2022 (5)	0.001800000	0.001522819	0.002077181
Kidane et al., 2016 (6)	0.000928302	0.000812299	0.001044305
	0.001209880	0.000462891	0.001956869

Statistics for each study; Rate and 95% CI

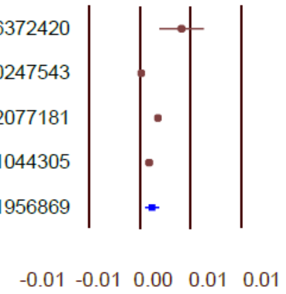

Test of null: Z-value=3.175, P=0.002
Heterogeneity: Q-value (df)=196.277 (3), P= 0.000
I-squared = 98.472
Tau-squared = 0.000

b. Forest plot for incidence rate of rabies in dogs

Figs (a, b, c, d, & e): Forest plots for the incidence rates of rabies in animals

Incidence of suspected rabies in cats

Study name	Rate	Lower limit	Upper limit
Wakgari et al., 2022 (5)	0.000228007	0.000099002	0.000357011
Kidane et al., 2016 (6)	0.000056604	0.000027959	0.000085249
	0.000130291	-0.000036022	0.000296604

Statistics for each study; Rate and 95% CI

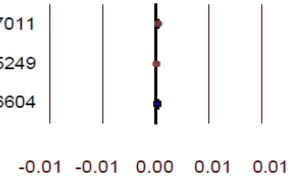

Test of null: Z-value=1.535, P=0.125
Heterogeneity: Q-value (df) = 6.463 (1), P= 0.011
I-squared = 84.527
Tau-squared = 0.000

c. Forest plot for incidence rate of rabies in cats

Incidence of suspected rabies in equines

Study name	Rate	Lower limit	Upper limit
Jemberu et al., 2013 (2)	0.000676786	0.000175425	0.001178147
Menghistu et al., 2018 (4)	0.000016173	0.000004192	0.000028153
Wakgari et al., 2022 (5)	0.000236998	0.000167753	0.000306243
	0.000195665	-0.000018450	0.000409779

Statistics for each study; Rate and 95% CI

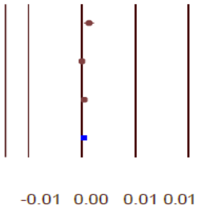

Test of null: Z-value= 1.791, P=0.073
Heterogeneity: Q-value (df) = 44.469 (2), P= 0.000
I-squared = 95.503
Tau-squared = 0.000

d. Forest plot for incidence rate of rabies in equines

Incidence of suspected rabies in goats

Study name	Rate	Lower limit	Upper limit
Jemberu et al., 2013 (2)	0.000144488	-0.000055758	0.000344734
Wakgari et al., 2022 (5)	0.000037000	0.000017618	0.000056381
	0.000042643	-0.000004343	0.000089629

Statistics for each study; Rate and 95% CI

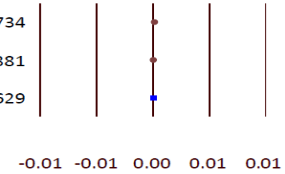

Test of null: Z-value= 1.779, P=0.075
Heterogeneity: Q-value (df)= 1.097 (1), P= 0.295
I-squared = 8.807
Tau-squared = 0.000

e. Forest plot for incidence rate of rabies in goats

References

1. Alemu YF, Jemberu WT, Mekuriaw Z, Abdi RD. Incidence and Predictors of Calf Morbidity and Mortality From Birth to 6-Months of Age in Dairy Farms of Northwestern Ethiopia. Frontiers in Veterinary Science. 2022;9.
2. Jemberu WT, Molla W, Almaw G, Alemu S. Incidence of Rabies in Humans and Domestic Animals and People's Awareness in North Gondar Zone, Ethiopia. PLoS Negl Trop Dis. 2013; 7(5).
3. Jibat T, Mourits MC, Hogeveen H. Incidence and economic impact of rabies in the cattle population of Ethiopia. Prev Vet Med. 2016; 130: 67-76.
4. Menghistu HT, Hailu KT, Shumye NA, Redda YT. Mapping the epidemiological distribution and incidence of major zoonotic diseases in South Tigray, North Wollo and Ab'ala (Afar), Ethiopia. PLoS One. 2018; 13 (12).
5. Wakgari M, Getachew G, Van't Klooster G, Tewolde N, Kivaria F, Bebay C. Participatory epidemiological study on the burden of rabies in animals and humans in three districts of Buno Bedele Zone, West Ethiopia. Ethiopian Veterinary Journal. 2022; 26 (2):93-106.
6. Kidane A, Sefir D, Bejiga T, Deressa A, Pal M. Rabies in animals with emphasis on dog and cat in Ethiopia. Worlds Veterinary Journal. 2016; 6 (1):123.
