# Supplementary material for: Incidence, risk factors, and control of Rabies in Ethiopia: A systematic review and meta-analysis
Source: PLoS Negl Trop Dis. 2025 Mar 19;19(3):e0012874. doi: 10.1371/journal.pntd.0012874 (PMC11922250; doi:10.1371/journal.pntd.0012874)
Supplement: Attachment — Submitted filename: PNTD-D-24-00387 (2)_With Watermark.pdf [file pntd.0012874.s004.pdf]

PLOS Neglected Tropical Diseases

Incidence, risk factors, and control of Rabies in Ethiopia: A systematic review and meta-analysis

--Manuscript Draft--

**Manuscript Number:**	PNTD-D-24-00387
**Full Title:**	Incidence, risk factors, and control of Rabies in Ethiopia: A systematic review and meta-analysis
**Short Title:**	Incidence, risk factors, and control of Rabies in Ethiopia
**Article Type:**	Research Article
**Keywords:**	Control; Ethiopia; Incidence; Rabies; Risk factors
**Abstract:**	Background<br>Globally, rabies is found in several geographical areas, with tens of thousands of deaths annually, mainly in developing countries. However, though Ethiopia is highly endemic for rabies, the overall risk of rabies has not yet been estimated. Hence, this systematic review and meta-analysis aimed at estimating a pooled incidence rate of human exposure to suspected rabid animals as well as the incidence rates of rabies in humans and other domestic animals.<br>Methods<br>A published article search was systematically performed through PubMed, Scopus, Embase, and Web of Science databases to identify the available studies on rabies until October 2023. The Joanna Brigg's Institute (JBI) critical appraisal checklists were used for assessing the quality of the studies. The PRISMA 2020 guideline was followed. A qualitative synthesis was made describing the characteristics of the included studies. The quantitative synthesis was performed with a random effects model using Comprehensive Meta-Analysis (CMA) version 3.0 software. The Q statistic quantified by I2 was used to check for heterogeneity among the included studies. To explain the source of heterogeneity, subgroup analysis was performed. Egger's regression test was used to evaluate publication biases. This study is registered with PROSPERO, CRD42023468791.<br>Results<br>For this study, a total of 439 articles were retrieved; of which fifteen studies were included in the final review. The annual pooled incidence rate of human exposure to suspected rabid animals was 33.65 (95% CI: 31.82 to 35.49) per 100,000 humans. The suspected rabies deaths in humans were also estimated to be 4.57 (95% CI: 2.93 to 6.21) per one million humans annually. In both cases, considerable heterogeneities were presented across the included studies, and obvious publication biases were detected using Egger's regression test. Among animals, the highest combined estimate per 100,000 population was recorded in dogs, 120.99 (95% CI: 46.29 to 195.69), followed by equines and cattle, with pooled incidence rates of 19.57 (95% CI: -1.85 to 40.98) and 18.08 (95% CI: 1 to 35.15), respectively. It was also described that human exposure to rabid animals and human rabies deaths were more common among children.<br>Conclusions<br>The current study showed a high pooled incidence rate of human exposure to rabid animals. Significant overall incidence rates of rabies in humans and animals were also indicated. Therefore, strengthening intersectoral and transdisciplinary collaborations through one health approach are key components for rabies prevention and control.
**Additional Information:**	
**Question**	**Response**
**Government Employee**<br><br>Are you or any of the contributing authors an employee of the United States government?	No - No authors are employees of the U.S. government.

Powered by Editorial Manager® and ProduXion Manager® from Aries Systems Corporation

Manuscripts authored by one or more US Government employees are not copyrighted, but are licensed under a CC0 Public Domain Dedication, which allows unlimited distribution and reuse of the article for any lawful purpose. This is a legal requirement for US Government employees.<br><br>This will be typeset if the manuscript is accepted for publication.	
**Financial Disclosure**<br><br>Enter a financial disclosure statement that describes the sources of funding for the work included in this submission. Review the submission guidelines for detailed requirements. View published research articles from *PLOS Neglected Tropical Diseases* for specific examples.<br><br>This statement is required for submission and **will appear in the published article** if the submission is accepted. Please make sure it is accurate.<br><br>**Funded studies**<br>Enter a statement with the following details:<br>• Initials of the authors who received each award<br>• Grant numbers awarded to each author<br>• The full name of each funder<br>• URL of each funder website<br>• Did the sponsors or funders play any role in the study design, data collection and analysis, decision to publish, or preparation of the manuscript?<br><br>Did you receive funding for this work?	The author(s) received no specific funding for this work.
**Competing Interests**<br><br>On behalf of all authors, disclose any competing interests that could be perceived to bias this work.	TThe authors have declared that no competing interests exist.

This statement will be typeset if the manuscript is accepted for publication.<br><br>Review the instructions link below and PLOS NTDs' competing interests policy to determine what information must be disclosed at submission.	
**Data Availability**<br><br>Provide a **Data Availability Statement** in the box below. This statement should detail where the data used in this submission can be accessed. This statement will be typeset if the manuscript is accepted for publication.<br><br>Before publication, authors are required to make all data underlying their findings fully available, without restriction. Review our PLOS Data Policy page for detailed information on this policy. Instructions for writing your Data Availability statement can be accessed via the Instructions link below.	All relevant data are within the paper and its supporting information files.

Click here to access/download;Manuscript;Manuscript on rabies-SR and MA.docx

Incidence, risk factors, and control of Rabies in Ethiopia: A systematic review and meta-analysis

Belay Tafa Regassa[1*], Wagi Tosisa[1], Daniel Eshetu[2], Andargachew Mulu[3], Gadissa Bedada Hundie[4]

[1]Department of Medical Laboratory Sciences, College of Health Sciences and Referral Hospital, Ambo University, Ambo, Ethiopia

[2]Department of Microbiology, Yirgalem Hospital Medical College, Sidama Regional State, Yirgalem, Ethiopia

[3]Armauer Hansen Research Institute, Addis Ababa, Ethiopia

[4]Department of Microbiology, Immunology & Parasitology, St. Paul's Hospital Millennium Medical College, Addis Ababa, Ethiopia

*Corresponding author: Belay Tafa Regassa; E-mail: belaytf@gmail.com

Abstract

Background

35- rephrase considering plural

Globally, rabies is found in several geographical areas, with tens of thousands of deaths annually mainly in developing countries. However, though Ethiopia is highly endemic for rabies, the overall risk of rabies has not yet been estimated. Hence, this systematic review and meta-analysis aimed at estimating a pooled incidence rate of human exposure to suspected rabid animals as well as the incidence rates of rabies in humans and other domestic animals.

Methods

A published article search was systematically performed through PubMed, Scopus, Embase, and Web of Science databases to identify the available studies on rabies until October 2023. The Joanna Brigg's Institute (JBI) critical appraisal checklists were used for assessing the quality of the studies. The PRISMA 2020 guideline was followed. A qualitative synthesis was made describing the characteristics of the included studies. The quantitative synthesis was performed with a random effects model using Comprehensive Meta-Analysis (CMA) version 3.0 software. The Q statistic quantified by I^2 was used to check for heterogeneity among the included studies. To explain the source of heterogeneity, subgroup analysis was performed. Egger's regression test was used to evaluate publication biases. This study is registered with PROSPERO, CRD42023468791.

Results

For this study, a total of 439 articles were retrieved; of which fifteen studies were included in the final review. The annual pooled incidence rate of human exposure to suspected rabid animals was 33.65 (95% CI: 31.82 to 35.49) per 100,000 humans. The suspected rabies deaths in humans were also estimated to be 4.57 (95% CI: 2.93 to 6.21) per one million humans annually. In both cases, considerable heterogeneities were presented across the included studies, and obvious publication biases were detected using Egger's regression test. Among animals, the highest combined estimate per 100,000 population was recorded in dogs, 120.99 (95% CI: 46.29 to 195.69), followed by equines and

Wondershare PDFelement

cattle, with pooled incidence rates of 19.57 (95% CI: -1.85 to 40.98) and 18.08 (95% CI: 1 to 35.15), respectively. It was also described that human exposure to rabid animals and human rabies deaths were more common among children.

Conclusions

The current study showed a high pooled incidence rate of human exposure to rabid animals. Significant overall incidence rates of rabies in humans and animals were also indicated. Therefore, strengthening intersectoral and transdisciplinary collaborations through one health approach are key components for rabies prevention and control.

Author Summary

Rabies virus infects all mammals with a fatality rate of nearly 100%. More than 99% of human rabies cases are transmitted via dog bites. Becoming one of the neglected tropical diseases, rabies occurs in several geographical areas. In Ethiopia, rabies vaccination coverage among dogs is far below the optimal 70%. This is due to the limited anti-rabies vaccination, lack of awareness among dog owners, and a high number of stray dogs. However, the overall risk of rabies has not yet been estimated in the country. Information on rabies incidence is important for notifying the population at risk and for planning effective prevention, control, and elimination strategies. We performed a systematic review and meta-analysis of published studies in Ethiopia. The annual pooled incidence rate of human exposure to suspected rabid animals was 33.65 per 100,000 humans. The suspected rabies deaths in humans were also estimated to be 4.57 per one million humans annually. Among animals, the highest combined estimate per 100,000 populations was recorded in dogs, 120.99, followed by equines and cattle, with pooled incidence rates of 19.57 and 18.08, respectively. We also found that human exposure to rabid animals and human rabies deaths were more common among children.

Wondershare PDFelement

Introduction

Rabies virus infects all mammals, including humans, with a fatality rate of nearly 100% once the clinical symptoms appear. Rabies occurs in more than 150 countries and almost half of the global population is at risk of contracting rabies living in canine rabies-endemic areas. It causes tens of thousands of deaths every year, becoming one of the neglected zoonoses, mainly in Asia and Africa (1, 2). More than 99% of human rabies cases are transmitted via dogs (1). It has also been recorded that human-to-human transmission of rabies is possible (3). Rabies has several impacts globally, including an impact on the budget of public health sectors, local communities, and livestock economies, where the poorest regions of the world are most affected. On average, it has been estimated that canine rabies causes approximately 59,000 human deaths, over 3.7 million disability-adjusted life years (DALYs), and more than 8.5 billion USD in economic losses worldwide annually (4).

As a zoonotic disease, rabies requires close cross-sectoral coordination of activities at the national, regional, and global levels. Rabies is included in the World Health Organization's (WHO's) 2021–2030 Roadmap for the Global Control of Neglected Tropical Diseases, which sets regional, progressive targets for the elimination of targeted diseases (2). Evidence shows that canine rabies has been eliminated in certain countries, like Western Europe, Canada, the USA, Japan, a few Latin American countries, and many parts of Asia. However, rabies is still widely occurring in over 80 countries, particularly in low and middle-income countries (1, 4), like Ethiopia. Analysis of the existing evidence showed that Ethiopia is still in the early stages of rabies control (5).

The large dog population and their poor management contribute to the high endemicity of canine rabies in Ethiopia (6). According to the CDC, rabies results in the death of 2,700 persons annually in Ethiopia, making it one of the highest death rates in the world (7). Furthermore, extrapolation of the district data to the national level indicated an annual estimate of approximately 3,000 human deaths, 194,000 DALYs, and 97,000 exposed persons, requiring on average 2 million USD in treatment costs per year countrywide (8). However, the true number of deaths caused by rabies is unknown in Ethiopia

93- contributes

paragraph 93 shoud discuss dog management as stated in the first line

Wondershare PDFelement

because the disease is underreported and rabies diagnostic laboratories are not widely established; only one national reference laboratory exists in the country (7).

Rabies is 100% preventable in animals and humans through vaccination (9), and hence, dog vaccination is considered the most cost-effective strategy for preventing rabies in humans (2). However, rabies vaccination coverage among dogs is very low in Ethiopia, far below the optimal 70% required to halt the transmission of canine rabies (7). This is partly due to the limited anti-rabies vaccination given to a small number of dogs found in urban settings (10). The others are a lack of awareness about rabies vaccination among dog owners and a high number of stray dogs in the country (7). According to the WHO, pre-exposure prophylaxis is recommended for those with a continued, frequent, or increased risk of exposure to the rabies virus, for example, veterinarians, laboratory workers, and animal handlers. In cases where individuals are exposed to a rabid animal, immediate vaccination and the administration of rabies immunoglobulin for high-risk exposures are indicated (2). However, few places in Ethiopia offer life-saving human rabies post-exposure prophylaxis (PEP). In addition, people’s poor awareness about what to do if bitten by dogs means that they often do not seek medical help when bitten (7).

The annual livestock losses caused by rabies place a large societal and economic burden on the country, although the total number of animal rabies cases in Ethiopia is unknown. In general, a high rabies burden and low vaccination status were recorded among dogs inflicting bites in Addis Ababa, the capital of Ethiopia. A low proportion of dogs, only 27%, were vaccinated and laboratory-confirmed rabid dogs were 70.3%, which is alarming, with seven out of ten dogs diagnosed as being infected with rabies (11). In humans and other animals, the overall prevalence of rabies was estimated to be 32% in Ethiopia (12). However, the overall risk of rabies in a population has not yet been estimated in the country. Information on the rabies incidence rate is important for notifying the population at risk of the disease and for planning effective prevention, control, and elimination strategies.

Hence, this systematic review and meta-analysis aimed at providing an overall estimate of the incidence rate of human exposure to suspected rabid animals as well as the incidence rates of rabies in

Wondershare PDFelement

humans and other domestic animals. Furthermore, it explored the associated risk factors of human rabies, its exposures, and control measures based on the published literature.

Methods

Databases and search strategies

The review protocol has been registered with the registration number CRD42023468791 in the International Prospective Register of Systematic Reviews (PROSPERO). A search was comprehensively performed through PubMed, Scopus, Embase, and Web of Science databases. Keywords, medical subject headings (MeSH), and related terms were included in our search strategies. The search terms and strings for databases were provided as supplementary information (**Supplementary Information-I**). We have also searched for grey literature through grey literature reports, worldwide science, and Ethiopian universities as well as research institute repositories. Further, we searched articles from reference lists of included studies and related systematic reviews and meta-analyses through African Journal Online (AJOL) and Google Scholar.

Inclusion and exclusion criteria

The inclusion and exclusion criteria were predefined and used to screen titles and abstracts and evaluate full texts for eligibility. Observational studies, including cross-sectional and cohort (both retrospective and prospective) studies, were included. Studies addressing the incidence, risk factors, and/or control of rabies in humans and/or animals from Ethiopia were considered. Clinically or laboratory-confirmed cases of rabies as well as a history of dog or animal bites as sources of rabies exposure were considered. English-language literature and studies conducted in Ethiopia until October 6, 2023, from the time of the inception of each database, were included. On the other hand, all original articles outside of Ethiopia, review papers, books, letters, brief reports, case reports, and poster presentations were excluded during the screening of titles and abstracts. Articles with unrelated

outcome measures and those with missing or insufficient outcome(s) were also excluded during the screening of the full text.

Study selection and quality assessment

Firstly, all retrieved studies were imported to the Endnote X9 citation manager. Duplicated studies were automatically removed from the Endnote. According to the inclusion and exclusion criteria, two reviewers independently screened all articles retrieved by the search strategy by title and abstract for eligibility. In the process, any discrepancies between the reviewers were discussed and resolved jointly to reach a consensus, and, if necessary, the full text was accessed for further clarification. The full texts of the eligible articles were then retrieved and assessed by the two reviewers. Following the full-text review, two independent investigators assessed the quality of the studies using the Joanna Brigg's Institute (JBI) critical appraisal tools adapted for observational studies. In the case of discrepancies, the two reviewers discussed and resolved the issues. Otherwise, the decision of the third reviewer was accepted. Accordingly, studies with positive responses ("yes") for greater than or equal to half (≥50%) of the number of checklist items were graded as good quality articles and those with less than half (<50%) scores were considered poor quality articles.

Data Extraction and Management

Data extraction was performed on an Excel spreadsheet and included the following: study first author and year of publication, study design, study setting (community, health facility, or both), study population (humans, animals, or both), number of new cases during follow-up, the total number of the at-risk population, risk factors, control measures, and administrative regions or cities of Ethiopia. Furthermore, person/animal-year and annual incidence rates of rabies/rabies exposures were computed from the available data and included in the data extraction format (**Supplementary Information-II**). Extractions were done by two independent reviewers, and if disagreements happened during the data

extraction process, they were resolved through joint discussions between the reviewers. The data were used only once if the results were published multiple times.

Strategy for data synthesis

The PRISMA 2020 guideline was followed for a systematic review and meta-analysis (13). A flow diagram was used to illustrate the literature search and article selection processes (Figure 1). A table was also used to provide an overview of the characteristics of the included articles. The outcomes of the study were the combined incidences of the conditions of interest among the target population categories (incidences of rabies exposure or rabies in humans, incidence of rabies in dogs, and other animal species). A qualitative synthesis was made describing the characteristics of the included studies. The quantitative synthesis was performed with a random effects model. Comprehensive Meta-Analysis (CMA) Version 3.0 statistical software was used for meta-analysis so that an overall summary effect estimate of the incidence across studies was determined. Further, we checked for heterogeneity across studies and also performed subgroup analysis to identify the sources of heterogeneity. Heterogeneity was evaluated by the chi-square test on Cochrane's Q statistic, which was quantified by I^2 values. The I^2 statistic estimates the percentage of total variation across studies due to true between-study differences rather than chance. Sensitivity analyses were conducted to check the stability of the summary estimate, and publication biases were evaluated using Egger's regression test.

Results

Review processes and findings

Four databases were accessed and a total of 439 articles (PubMed = 80; Scopus = 109; Embase = 140; and Web of Science = 110) were found without limiting when publications started until October 6, 2023. After removing the duplicates (n = 224) using the Endnote reference manager, 215 records were kept. After screening for titles and abstracts, 157 irrelevant studies were removed, and 58 full-text papers were retrieved and assessed for eligibility. Of the 58 full-text papers, because of different

reasons, 49 articles were excluded, and 9 were found relevant. In addition to the databases, records were also sought from other sources such as free web search engines (African Journal Online and Google Scholar) and Grey Literature, and as a result, we obtained 28 full-text records. Assessing for eligibility, 22 records were excluded with reasons, while 6 were found relevant for the review. According to the quality assessment tool, all the included studies had good quality scores. Finally, 15 and 14 relevant articles were included in the systematic review and meta-analysis, respectively (Figure 1).

state the reasons for exclusion-197

state the scoring category and the criteria

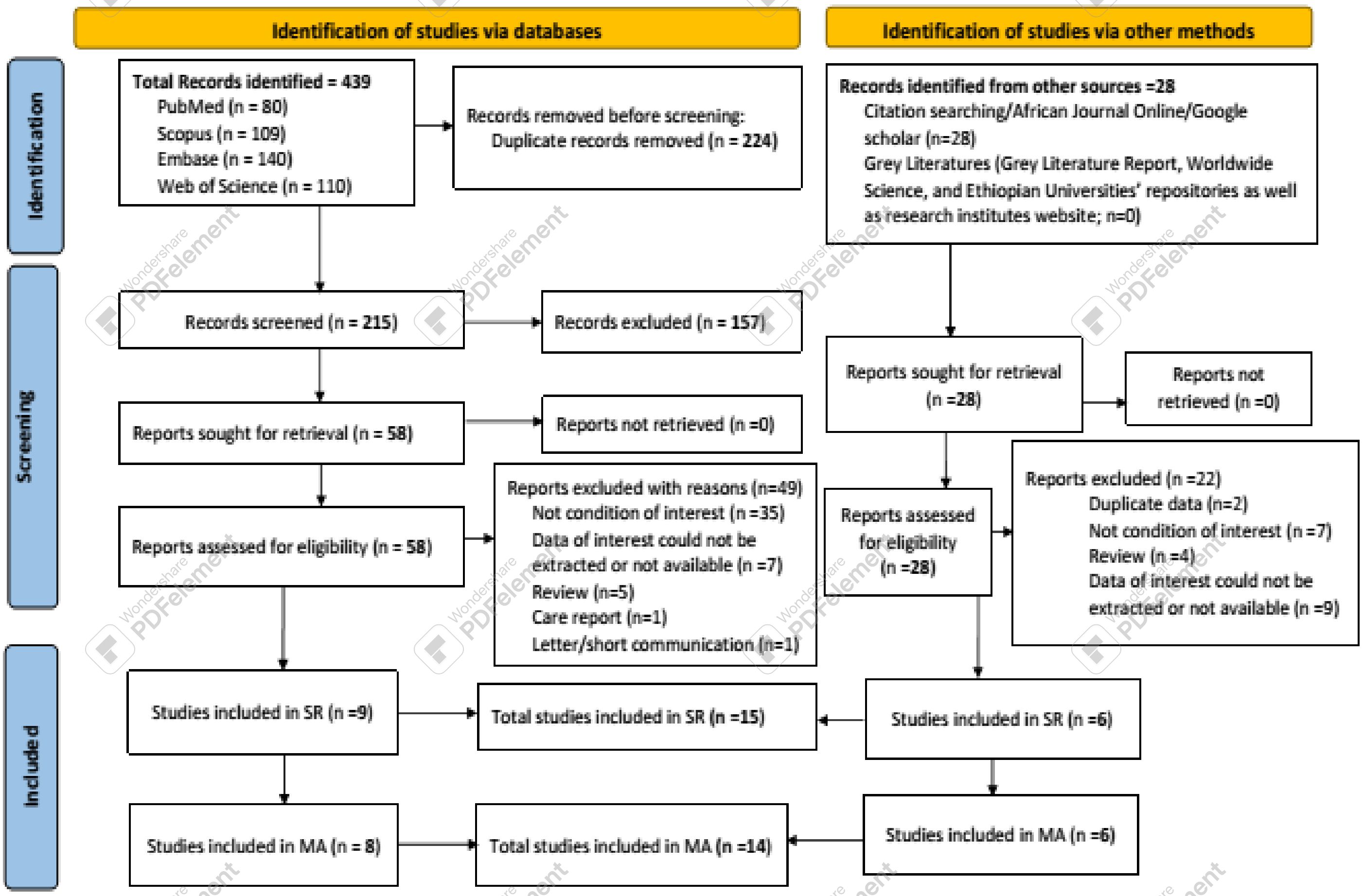

Figure 1 PRISMA 2020 Flow diagram for literature search and selection

Characteristics of the included studies

Describe the articles by year of publication

The study records were reported from four regional states and one city administration in Ethiopia. By region, 5 studies (33.3%) were from Oromia (14-18), 4 (26.7%) were from Amhara (19-22), 3 (20.0%) from Tigray (23-25), and one (6.7%) study was multi-regional (26) reporting aggregate data from three regions (Afar, Amhara and Tigray), while 2 (13.3%) studies were from Addis Ababa City

Administration (27, 28). The majority, 80% (12/15), of included studies employed a cross-sectional study design (14-18, 21-23, 25-28) where almost all of them used retrospective timing frames. The target study population was humans in 60% (9/15) of the included studies (14, 16, 17, 21-25, 28), while it was 20% (3/15) each for animals (15, 19, 27) and both humans and animals (18, 20, 26). Regarding the settings used for conducting the studies, health facilities, animal clinics, and the community were utilized. More than half (53.3%) of the studies were conducted in or utilized records of health facilities only (16, 17, 21-25, 28). One or more outcomes were reported from each of the included studies, giving a total frequency of 29 outcomes: nine studies reported the incidence of human exposure to suspected rabid animals; three outcomes, such as the incidence of suspected rabies in humans and rabies in animals, and risk factors for exposures or rabies, were documented in 6 (20.7%) studies each; and the other 2 (6.9%) studies reported prevention/control measures taken to prevent rabies (Table 1).

Table 1 Profiles of studies included in the systematic review and meta-analysis

Study profiles		Included studies Number (%)
**Study design**	Cross-sectional	12 (80)
	Cohort	3 (20)
**Study setting**	Health facility only	8 (53.3)
	Community only	3 (20.0)
	Health facilities and community	1 (6.7)
	Health facility and animal clinic	2 (13.3)
	Animal clinic only	1 (6.7)
**Target population**	Humans	9 (60)
	Animals	3 (20)
	Both	3 (20)
**Region**	Oromia	5 (33.3)
	Amhara	4 (26.7)
	Tigray	3 (20.0)
	Addis Ababa	2 (13.3)
	Multi-regional (Afar, Amhara, Tigray)	1 (6.7)
**Outcomes reported***	Incidence of human exposure to suspected rabid animals	9 (30.0)
	Incidence of suspected rabies in humans	6 (20.7)
	Incidence of suspected rabies in animals	6 (20.7)
	Risk factors for rabies exposure or rabies	6 (20.7)
	Prevention/control measures for rabies	2 (6.9)

A health facility refers to a hospital or health center.
*For a single study, one or more outcome measures were reported.

Wondershare PDFelement

Number of studies/datasets included in systematic review and meta-analysis

In total, 15 studies were considered for the systematic review (14-28). Of these, 14 (93.3%) were included in the meta-analysis, where different numbers of studies and datasets were used for the different outcomes reported. Nine studies with 23 datasets were used in the meta-analysis for the incidence of human exposure to suspected rabid animals (14, 17, 20-25, 28), while 6 studies with 14 datasets were included for the incidence of suspected rabies in humans (14, 18, 20, 23, 26, 28). For the incidence of suspected rabies by animal types such as goats (18, 20), cats (18, 27), equines (18, 20, 26), dogs (18, 20, 26, 27), and cattle (15, 18-20, 26), 2 to 5 studies were included in the meta-analysis. Since the incidence of suspected rabies in camels was reported by one study (26), with a single dataset, it was not considered for meta-analysis. Moreover, 6 and 2 studies reporting the risk factors for rabies exposure or rabies deaths (16, 17, 20, 21, 25, 26) and control measures (17, 25) were included in the systematic review, respectively (Table 2).

Table 2 Studies and datasets included in systematic review and meta-analysis by outcome

**The outcome measured or reported by studies**		**Number of studies included in SR**	**Studies included in the MA**	
			Number of Studies	Number of datasets used
**Incidence of human exposure to suspected rabid animals**		9	9	23
**Incidence of suspected rabies in humans**		6	6	14
**Incidence of suspected rabies by animal type**	cattle	5	5	5
	dogs	4	4	4
	cats	2	2	2
	equines	3	3	3
	goats	2	2	2
	camels	1	NA	-
**Risk factors for rabies exposure or rabies**		6	-	-
**Prevention/control measures**		2	-	-

NA = Not applicable.

Wondershare PDFelement

Incidences of human exposure to rabid animals and human rabies from included studies

Overall, 11, 294 human exposures to suspected rabid animals were reported from the included studies. The reported incidence rates of human exposures ranged from zero in 2015 (28), to 422 per 100,000 humans in 2013 (23). In general, the highest incidences of human exposure to suspected rabid animals were reported in the Tigray region, ranging from 278 to 422 per 100,000 populations (23). Relatively, maximum incidences of exposures of 108.64 per 100,000 (14) and 200.64 per 100,000 (17) were also reported from Oromia regional state. The smallest estimates of incidences of exposures were reported from Addis Ababa and North Gondor, ranging from 0 to 1.97 (28) and 1.27 to 4.6 (21), respectively. However, higher incidences of exposures, 12.41 and 24.8 per 100,000, were recorded in the datasets from Addis Ababa (28), which were higher than the reports from Bahir Dar and other zones (22) (Table 3).

Regarding suspected rabies in humans, a total of 303 deaths were reported from different corners of Ethiopia, as indicated in the included studies. The annual incidence rate of human rabies recently reported from the Buno Bedele zone of Oromia was 4.19 per 100,000 population (18). A multi-regional record from three regions, Afar, Amhara, and Tigray, also reported that the incidence rate of rabies in humans was 4.13 per 100,000 populations (26). Annually, incidence rates of human rabies ranging from 1 to 2.94 per 100,000 populations were also reported in other studies (14, 20, 23). On the other hand, rare occurrences (≤ 1 per million) of suspected human rabies were recorded in some datasets from Addis Ababa (28) (Table 3).

Wondershare PDFelement

Table 3 Incidence rates of human exposure to rabid animals and human rabies from included studies

Outcome	Studies	Dataset	Annual incidence rate per 100,000 population	Region or City
**Human exposure to suspected rabid animals**	Beyene *et al*., 2018	-	108.64	Oromia
	Gebru *et al*., 2019	-	40.00	Tigray
	Jemberu *et al*., 2013	-	24.94	Amhara
	Ramos *et al*., 2015	-	200.64	Oromia
	Teklu *et al*., 2017	2012 data	35.83	Tigray
		2013 data	62.96	
		2014 data	89.75	
		2015 data	73.1	
	Yibrah & Damtie, 2015	2011 data	4.6	Amhara
		2012 data	2.61	
		2013 data	1.27	
	Yizengaw *et al*., 2018	2015/16 data	6.48	Amhara
		2016/17 data	7.54	
	Ebuy *et al*., 2019	2012 data	392	Tigray
		2013 data	422	
		2014 data	278	
		2015 data	308	
		2016 data	317	
	Mesfin, 2022	2015 data	0	Addis Ababa
		2016 data	0.1	
		2017 data	1.97	
		2018 data	12.41	
		2019 data	24.8	
**Suspected rabies in humans**	Beyene *et al*., 2018	-	2.8	Oromia
	Jemberu *et al*., 2013	-	2.34	Amhara
	Menghistu *et al*., 2018	-	4.13	Multi-regional (Afar, Amhara, Tigray)
	Ebuy *et al*., 2019	2012 data	2.94	Tigray
		2013 data	0.98	
		2014 data	1.11	
		2015 data	2.75	
		2016 data	1.68	
	Mesfin, 2022	2015 data	0	Addis Ababa
		2016 data	0	
		2017 data	0.12	
		2018 data	0.02	
		2019 data	0.04	
	Wakgari *et al*., 2022	-	4.19	Oromia

Wondershare PDFelement

Incidence rates of rabies in animals from included studies

Some studies reported the incidence rates of rabies in various domestic animals (Table 4). Among animals, the first and second highest estimates of suspected incidence per 100,000 animals, 1880.19 (15) and 911.16 (19), were reported in cattle, followed by that of dogs, 412.83 per 100,000 (20). The smallest incidence (0.3 per 100,000) was also reported in cattle (26). Relatively, other studies reported smaller estimates of rabies incidence in animals. All the included studies reported the incidence rates of suspected rabies, except one study (27) which reported the incidence of both suspected and laboratory-confirmed rabies in dogs and cats. Differences in the incidence rates per 100,000 populations were observed between suspected and confirmed cases of dogs (92.8 versus 70.2) and cats (5.7 versus 3.2); higher estimates were recorded for suspected animals (27). Only one study reported the incidence of rabies in camels, 0.69 per 100,000 populations (26).

Table 4 Incidence rates of suspected and confirmed rabies in animals from included studies

Animal type	Studies	Annual incidence rate per 100,000 population
**Cattle**	Alemu *et al.*, 2022	911.16
	Jemberu *et al.*, 2013	19.89
	Jibat *et al.*, 2016	1880.19
	Menghistu *et al.*, 2018	0.3
	Wakgari *et al.*, 2022	17.5
**Dogs**	Jemberu *et al.*, 2013	412.83
	Menghistu *et al.*, 2018	15.28
	Wakgari *et al.*, 2022	180
	Kidane *et al.*, 2016	92.8<br>70.2$^{\Omega}$
**Cats**	Wakgari *et al.*, 2022	22.8
	Kidane *et al.*, 2016	5.7<br>3.2$^{\Omega}$
**Equines**	Jemberu *et al.*, 2013	67.68
	Menghistu *et al.*, 2018	1.62
	Wakgari *et al.*, 2022	23.7
**Goats**	Jemberu *et al.*, 2013	14.45
	Wakgari *et al.*, 2022	3.7
**Camels**	Menghistu *et al.*, 2018	0.69

$^{\Omega}$ Laboratory confirmed cases; otherwise, suspected cases are based on clinical definition.

Risk factors of exposure to rabid animals or rabies in humans, and control measures

Discuss each risk factor sepertely. e.g - Age ....sex... etc

A few numbers of socio-demographic features of humans were reported as risk factors for the occurrence of exposure to suspected rabid animals (16, 17, 20, 21, 25, 26). Studies from Amhara and Tigray regional states reported that male sex was more associated with human exposure to suspected rabid animals (21, 25). Regarding the age of exposed humans to suspected rabid animals, two separate studies from Jimma and Arsi, Oromia, reported that children <15 years of age were much more likely exposed (16, 17). On the contrary, a study from the northwestern Tigray region reported that adult populations aged ≥15 years were most exposed (25). As to the residence, rural was recorded as the major factor for exposure to rabid animals from Amhara and Oromia (16, 20). However, another study from Amhara reported that urban residents were more affected than rural ones (21).

Concerning the risk factors for rabies mortality in humans, female sex and the age of 5-14 years were identified as risk factors (26). Dog bites were the source of exposure for all rabies mortality, as indicated by one study (20). From the records collected for review purposes, a limited number of studies reported what measures were taken once humans were exposed to suspected rabid animals or the status of vaccination of dogs/animals inflicting bites. One study from the west Arsi zone of Oromia reported that all the exposed individuals to rabid animals received nerve tissue vaccines, but only 1% of cases did not complete the vaccination course (17). On the other side, a study from northwestern Tigray published information that all the dogs inflicting unprovoked bites were unvaccinated (25).

Pooled incidence rate of human exposure to suspected rabid animals

The overall pooled incidence rate of human exposure to suspected rabid animals per 100,000 humans annually was 33.65 (95% CI: 31.82 to 35.49). Considerable heterogeneity was presented for the overall combined effect size (Q = 11222.628 (df = 22), *P*<0.001; I-squared = 99.804%). Of note, the overall true effect size was greater than zero (Z-value = 35.92, *P*<0.001) (Figure 2).

Incidence rate of human exposure to suspected rabid animals

Study name	Rate	Lower limit	Upper limit	Relative weight
Beyene et al., 2018	0.001086449	0.000987700	0.001185197	2.30
Ebuy et al., 2019 (2012 data)	0.003919991	0.003648185	0.004191797	0.43
Ebuy et al., 2019 (2013 data)	0.004220008	0.003938457	0.004501558	0.40
Ebuy et al., 2019 (2014 data)	0.002779998	0.002607523	0.002952474	0.97
Ebuy et al., 2019 (2015 data)	0.003080000	0.002889768	0.003270233	0.82
Ebuy et al., 2019 (2016 data)	0.003169999	0.002967566	0.003372432	0.73
Gebru et al., 2019	0.000399927	0.000359066	0.000440787	5.13
Jemberu et al., 2013	0.000249715	0.000163195	0.000336235	2.72
Mesfin, 2022 (2015 data)	0.000000129	-0.000000229	0.000000487	6.87
Mesfin, 2022 (2016 data)	0.000000990	0.000000020	0.000001960	6.87
Mesfin, 2022 (2017 data)	0.000019687	0.000015452	0.000023922	6.85
Mesfin, 2022 (2018 data)	0.000124091	0.000113682	0.000134499	6.73
Mesfin, 2022 (2019 data)	0.000248040	0.000233635	0.000262445	6.60
Ramos et al., 2015	0.002006363	0.001855894	0.002156832	1.22
Teklu et al., 2017 (2012 data)	0.000358277	0.000316899	0.000399655	5.10
Teklu et al., 2017 (2013 data)	0.000629606	0.000575229	0.000683983	4.29
Teklu et al., 2017 (2014 data)	0.000897465	0.000833107	0.000961824	3.73
Teklu et al., 2017 (2015 data)	0.000730987	0.000673906	0.000788067	4.13
Yibrah and Damtie, 2015 (2011 data)	0.000046009	0.000038388	0.000053631	6.79
Yibrah and Damtie, 2015 (2012 data)	0.000026116	0.000020429	0.000031803	6.83
Yibrah and Damtie, 2015 (2013 data)	0.000012652	0.000008731	0.000016572	6.85
Yizengaw et al., 2018 (2015/2016 data)	0.000064803	0.000058627	0.000070978	6.82
Yizengaw et al., 2018 (2016/2017 data)	0.000075419	0.000068815	0.000082023	6.81
**Overall estimate**	0.000336512	0.000318150	0.000354874	

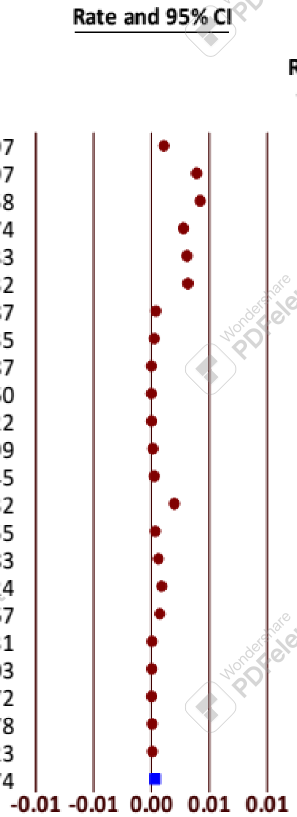

Heterogeneity: Q-value = 11222.628 (df = 22), P-value = 0.000
I-squared = 99.804
Tau-squared = 0.000
Test of null: z-value = 35.920, p-value = 0.000

Figure 2 Forest plot for the incidence rates of human exposure to suspected rabid animals

Subgroup analysis of incidence rates of human exposure to rabid animals by regions

In the meta-analysis of the incidence rates of human exposures to suspected rabid animals, substantial heterogeneity was found among the effect sizes of the included individual studies/datasets. Subgroup analysis was performed by regions of the studies/datasets to explore if they were the source of heterogeneity. The highest pooled incidence rate of human exposure to suspected rabid animals per 100,000 populations was recorded in Oromia, 138.91 (95% CI: 129.79 to 148.03), followed by Tigray regional state, 83.32 (95% CI: 80.25 to 86.39). The smallest pooled incidence by region was from Amhara, 5.52 (95% CI: 3.26 to 7.79), while it was 7.58 (95% CI: 5.25 to 9.91) from Addis Ababa. Considerable heterogeneities were also seen from the subgroup analysis between regions, indicating that regions of the studies would be sources of heterogeneity (Figure 3).

Incidence rate of human exposure to suspected rabid animals by regions in Ethiopia

Group by Region	Study name	Rate	Lower limit	Upper limit
Addis Ababa	Mesfin, 2022 (2015 data)	0.000000129	-0.000000229	0.000000487
Addis Ababa	Mesfin, 2022 (2016 data)	0.000000990	0.000000020	0.000001960
Addis Ababa	Mesfin, 2022 (2017 data)	0.000019687	0.000015452	0.000023922
Addis Ababa	Mesfin, 2022 (2018 data)	0.000124091	0.000113682	0.000134499
Addis Ababa	Mesfin, 2022 (2019 data)	0.000248040	0.000233635	0.000262445
Addis Ababa		0.000075791	0.000052476	0.000099106
Amhara	Jemberu et al., 2013	0.000249715	0.000163195	0.000336235
Amhara	Yibrah and Damtie, 2015 (2011 data)	0.000046009	0.000038388	0.000053631
Amhara	Yibrah and Damtie, 2015 (2012 data)	0.000026116	0.000020429	0.000031803
Amhara	Yibrah and Damtie, 2015 (2013 data)	0.000012652	0.000008731	0.000016572
Amhara	Yizengaw et al., 2018 (2015/2016 data)	0.000064803	0.000058627	0.000070978
Amhara	Yizengaw et al., 2018 (2016/2017 data)	0.000075419	0.000068815	0.000082023
Amhara		0.000055244	0.000032639	0.000077850
Oromia	Beyene et al., 2018	0.001086449	0.000987700	0.001185197
Oromia	Ramos et al., 2015	0.002006363	0.001855894	0.002156832
Oromia		0.001389141	0.001297911	0.001480370
Tigray	Ebuy et al., 2019 (2012 data)	0.003919991	0.003648185	0.004191797
Tigray	Ebuy et al., 2019 (2013 data)	0.004220008	0.003938457	0.004501558
Tigray	Ebuy et al., 2019 (2014 data)	0.002779998	0.002607523	0.002952474
Tigray	Ebuy et al., 2019 (2015 data)	0.003080000	0.002889768	0.003270233
Tigray	Ebuy et al., 2019 (2016 data)	0.003169999	0.002967566	0.003372432
Tigray	Gebru et al., 2019	0.000399927	0.000359066	0.000440787
Tigray	Teklu et al., 2017 (2012 data)	0.000358277	0.000316899	0.000399655
Tigray	Teklu et al., 2017 (2013 data)	0.000629606	0.000575229	0.000683983
Tigray	Teklu et al., 2017 (2014 data)	0.000897465	0.000833107	0.000961824
Tigray	Teklu et al., 2017 (2015 data)	0.000730987	0.000673906	0.000788067
Tigray		0.000833213	0.000802495	0.000863931

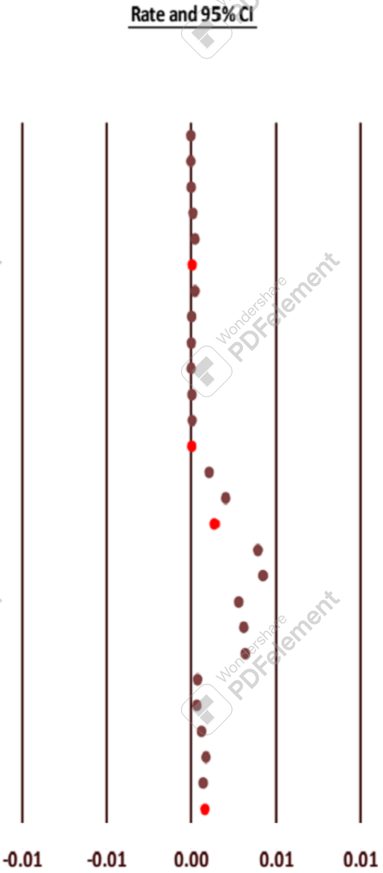

Region	Test of null (2-Tail)	Heterogeneity statistics		Tau-squared
		Q-value (df); P-value	I -squared	
Addis Ababa	Z = 6.371, P = 0.000	1759.503 (4); P=0.000	99.773	0.000
Amhara	Z = 4.790, P = 0.000	398.298 (5); P=0.000	98.745	
Oromia	Z = 29.844, P = 0.000	100.358 (1); P=0.000	99.004	
Tigray	Z =53.163, P = 0.000	3377.590 (9); P=0.000	99.734	
Total between	-	2481.082 (3); P=0.000	-	-

A random effects model was used to combine studies within each subgroup. The study-to-study variance (tau-squared) was assumed to be the same for all subgroups; this value is computed within subgroups and then pooled across subgroups.

Figure 3 Forest plot for subgroup analysis of incidences of human exposure by regions

Sensitivity analysis and publication bias for incidence of human exposure to rabid animals

Performing the sensitivity analysis by removing one study or dataset at a time, the overall results were noticeably changed when two datasets (datasets of 2015 and 2016 each) from the study reported by Mesfin (28) were omitted one by one, giving the pooled incidence rates of 64.84 (95% CI: 60.97 to 68.70) and 63.61 (95% CI: 59.85 to 67.37) per 100,000, respectively. Otherwise, the results were comparable with the overall effect when none of the studies were removed (Figure 4). To check for publication bias, Egger's regression test was performed, and obvious publication bias was detected

Wondershare PDFelement

among the publications that reported the incidence of human exposure to suspected rabid animals (Intercept = 22.06 (95% CI: 18.13 to 26.00), P<0.001).

Incidence rate of human exposure to suspected rabid animals with one study or dataset removed

Study name	Statistics with study removed: Point	Lower limit	Upper limit
Beyene et al., 2018	0.000313450	0.000295205	0.000331695
Ebuy et al., 2019 (2012 data)	0.000312062	0.000294228	0.000329896
Ebuy et al., 2019 (2013 data)	0.000311118	0.000293332	0.000328903
Ebuy et al., 2019 (2014 data)	0.000301517	0.000283782	0.000319252
Ebuy et al., 2019 (2015 data)	0.000302699	0.000284983	0.000320414
Ebuy et al., 2019 (2016 data)	0.000305083	0.000287328	0.000322839
Gebru et al., 2019	0.000328470	0.000309878	0.000347062
Jemberu et al., 2013	0.000338571	0.000319975	0.000357167
Mesfin, 2022 (2015 data)	0.000648389	0.000609733	0.000687045
Mesfin, 2022 (2016 data)	0.000636094	0.000598489	0.000673700
Mesfin, 2022 (2017 data)	0.000365105	0.000345782	0.000384428
Mesfin, 2022 (2018 data)	0.000345914	0.000327229	0.000364598
Mesfin, 2022 (2019 data)	0.000328248	0.000310044	0.000346452
Ramos et al., 2015	0.000308182	0.000290194	0.000326169
Teklu et al., 2017 (2012 data)	0.000331755	0.000313109	0.000350402
Teklu et al., 2017 (2013 data)	0.000317000	0.000298600	0.000335399
Teklu et al., 2017 (2014 data)	0.000305752	0.000287579	0.000323924
Teklu et al., 2017 (2015 data)	0.000311755	0.000293456	0.000330054
Yibrah and Damtie, 2015 (2011 data)	0.000357905	0.000338873	0.000376937
Yibrah and Damtie, 2015 (2012 data)	0.000361752	0.000342590	0.000380915
Yibrah and Damtie, 2015 (2013 data)	0.000367214	0.000347801	0.000386627
Yizengaw et al., 2018 (2015/2016 data)	0.000354063	0.000335171	0.000372954
Yizengaw et al., 2018 (2016/2017 data)	0.000351877	0.000333063	0.000370691
	0.000336512	0.000318150	0.000354874

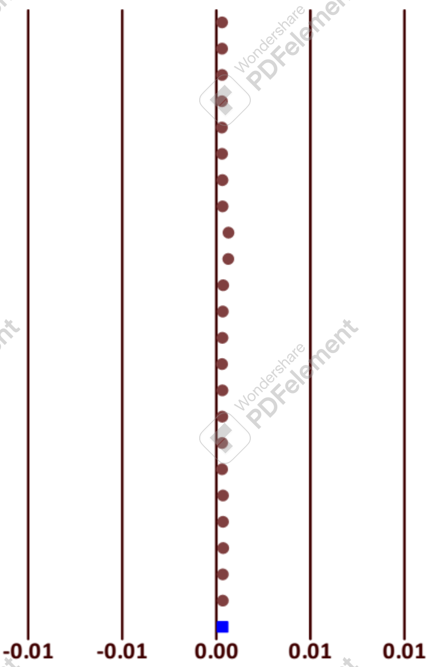

Figure 4 Forest plot of sensitivity analysis for incidence rates of human exposure to rabid animals

Pooled incidence rate of suspected human rabies

The annual pooled incidence rate of suspected human rabies was 4.57 (95% CI: 2.93 to 6.21) per one million humans. It was indicated that the overall true effect size was greater than zero (Z-value = 5.461, *P*<0.001). Considerable heterogeneity was presented for the overall combined effect size (Q = 295.141 (df = 13), *P*<0.001; I-squared = 95.595%) (Figure 5). Conducting sensitivity analysis, the overall results were not noticeably changed except in the case of removing one study reporting the incidence of human rabies by Menghistu and others (26) where the combined effect of the rest of the studies/datasets gave a very small pooled incidence of <1 per one million (Figure 6). Obvious publication bias was also detected using Egger's regression test among the publications that reported the incidence of human rabies (Intercept (B_o) = 4.01505 (95% CI: 1.43272 to 6.59738), P = 0.0027).

Wondershare PDFelement

Incidence rate of suspected human rabies

Study name	Rate	Lower limit	Upper limit	Z-Value	p-Value	Relative weight
Beyene et al., 2018	0.000028037	0.000012174	0.000043901	3.464101615	0.000532006	1.01
Ebuy et al., 2019 (2012 data)	0.000029437	0.000005883	0.000052991	2.449489743	0.014305878	0.47
Ebuy et al., 2019 (2013 data)	0.000009780	-0.000003774	0.000023334	1.414213562	0.157299207	1.35
Ebuy et al., 2019 (2014 data)	0.000011142	0.000000223	0.000022062	2.000000000	0.045500264	2.00
Ebuy et al., 2019 (2015 data)	0.000027527	0.000009543	0.000045511	3.000000000	0.002699796	0.79
Ebuy et al., 2019 (2016 data)	0.000016826	0.000002078	0.000031574	2.236067978	0.025347319	1.16
Jemberu et al., 2013	0.000023411	-0.000003081	0.000049902	1.732050808	0.083264517	0.38
Menghistu et al., 2018	0.000041251	0.000035885	0.000046617	15.066519173	0.000000000	6.10
Mesfin, 2022 (2015 data)	0.000000129	-0.000000229	0.000000487	0.707106781	0.479500122	17.42
Mesfin, 2022 (2016 data)	0.000000124	-0.000000219	0.000000467	0.707106781	0.479500122	17.43
Mesfin, 2022 (2017 data)	0.000001186	0.000000146	0.000002225	2.236067978	0.025347319	16.40
Mesfin, 2022 (2018 data)	0.000000227	-0.000000218	0.000000673	1.000000000	0.317310508	17.34
Mesfin, 2022 (2019 data)	0.000000436	-0.000000168	0.000001039	1.414213562	0.157299207	17.15
Wakgari et al., 2022	0.000041925	0.000026111	0.000057739	5.196152423	0.000000203	1.01
	0.000004570	0.000002930	0.000006210	5.461267568	0.000000047	

Rate and 95% CI

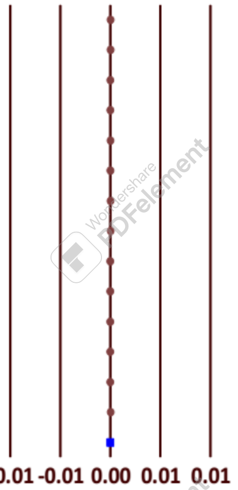

Heterogeneity: Q-value = 295.141 (df = 13), P-value = 0.000
I-squared = 95.595
Tau-squared = 0.000
Test of null: Z-value = 5.461, P-value = 0.000

Figure 5 Forest plot for the incidence rates of suspected human rabies

Incidence rate of suspected human rabies with one study or dataset removed

Study name	Point	Lower limit	Upper limit	Z-Value	p-Value
Beyene et al., 2018	0.000004236	0.000002616	0.000005857	5.122833672	0.000000301
Ebuy et al., 2019 (2012 data)	0.000004408	0.000002776	0.000006039	5.295047238	0.000000119
Ebuy et al., 2019 (2013 data)	0.000004491	0.000002842	0.000006140	5.337415151	0.000000094
Ebuy et al., 2019 (2014 data)	0.000004411	0.000002762	0.000006061	5.240744775	0.000000160
Ebuy et al., 2019 (2015 data)	0.000004317	0.000002690	0.000005943	5.200930865	0.000000198
Ebuy et al., 2019 (2016 data)	0.000004393	0.000002753	0.000006032	5.249637320	0.000000152
Jemberu et al., 2013	0.000004481	0.000002843	0.000006120	5.361298519	0.000000083
Menghistu et al., 2018	0.000000840	0.000000005	0.000001674	1.971378013	0.048680654
Mesfin, 2022 (2015 data)	0.000007129	0.000004909	0.000009349	6.294538952	0.000000000
Mesfin, 2022 (2016 data)	0.000007210	0.000004969	0.000009451	6.305495644	0.000000000
Mesfin, 2022 (2017 data)	0.000005413	0.000003575	0.000007251	5.772403119	0.000000008
Mesfin, 2022 (2018 data)	0.000006609	0.000004520	0.000008698	6.201614737	0.000000001
Mesfin, 2022 (2019 data)	0.000006040	0.000004085	0.000007995	6.055405740	0.000000001
Wakgari et al., 2022	0.000003968	0.000002386	0.000005549	4.917433824	0.000000877
	0.000004570	0.000002930	0.000006210	5.461267568	0.000000047

Rate (95% CI) with study removed

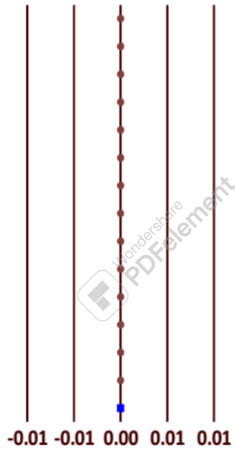

Figure 6 Forest plot for sensitivity analysis of incidence rate of suspected human rabies

Pooled incidence rate of suspected rabies in animals

The annual incidence rates of suspected rabies were pooled for different domestic animals in Ethiopia. The highest combined effect per 100,000 population was recorded in dogs, 120.99 (95% CI: 46.29 to 195.69), followed by equines, 19.57 (95% CI: -1.85 to 40.98), and cattle, 18.08 (95% CI: 1 to 35.15). The pooled incidence rates of rabies were 13.03 (95% CI: -3.60 to 29.66) and 4.26 (95% CI: -0.43 to 8.96) per 100,000 in cats and goats, respectively. However, the overall true effect sizes could not be different from zero in cats (Z-value = 1.535, *P* = 0.125), equines (Z-value = 1.791, *P* = 0.173), and goats (Z-value = 1.779, *P* = 0.075), as also indicated by the 95% CI of the pooled estimates in each. On the contrary, the true effect sizes were different from zero in dogs (Z-value = 3.175, *P* = 0.002) and cattle (Z-value = 2.071, *P*<0.001). Considerable heterogeneities were found for the overall combined effect sizes in all the animals except for goats, where no variation was revealed among the included studies (Q-value (df) = 1.097 (1), *P* = 0.295; I-squared = 8.807) (**Supplementary Information – III;** Figures a, b, c, d, & e).

Discussion

Rabies is one of the neglected zoonotic diseases, with half of the world's population living in rabies-endemic territories and at risk of contracting the virus. Every year, it accounts for tens of thousands of deaths, mainly in developing countries (1, 2). Although the WHO has targeted rabies for its elimination, including it in its 2021–2030 roadmap for the global control of neglected tropical diseases (2), Ethiopia is still in the early phase of rabies control (5).

Some individual studies on the incidence of rabies have been reported from some districts or regions of Ethiopia, which might not reveal the incidence of rabies in the entire country. The absence of summarized data on the incidence of rabies, both in humans and animals, might be among the major problems that cause negligent commitment to the control of rabies. Determination of the overall quantitative estimate is required in resource-limited settings, such as Ethiopia, to prioritize the prevention and control strategies specific to rabies because of numerous competing interests. As the

Wondershare PDFelement

overall estimate of rabies in the nation is important to attract the attention of policy-makers towards the allocation of sufficient resources for reducing human and animal rabies, pooled evidence on the incidence of rabies is lacking in Ethiopia to our current knowledge. Hence, this review aimed to provide the overall estimates of incidence rates of human exposure to suspected rabid animals and the incidence rate of rabies in humans as well as domestic animals based on the published research articles.

For synthesizing the current evidence, the records on the incidence of rabies and related outcomes were reported from four regional states, Oromia, Amhara, Tigray, and Afar (14-26), and one city administration, Addis Ababa (27, 28) of Ethiopia. This showed that the reports were from about one-third of the geographical administrations of the country, indicating that the problem at hand has not been equally addressed in different locations of the country. The majority of the studies targeted humans, utilizing cross-sectional study designs through retrospective data analysis of recorded information from health facilities. This implied that data from prospective types of studies and active surveillance systems are lacking or limited in the country. Compared with incidence studies conducted on humans, a relatively small number of studies were conducted on animals, though animals, especially dogs, are almost always the source of humans' and other animals' infections.

In the current review, the incidence rates of human exposures to suspected rabid animals from the incidence data of the included studies ranged from 0 to 422 (95% CI: 394 to 450) per 100,000 persons annually. This review revealed that estimates of human exposure to rabid animals were not uniform in the country, where the incidences varied widely across the regions as well as within the regions. However, the information can valuably alert the local or regional administrators to proactively plan for the prevention of rabies, such as purchasing optimal doses of post-exposure prophylaxis. The annual incidence rates of suspected human rabies from the datasets of the included studies ranged from 0 to 4.19 (95% CI: 2.61 to 5.77) per 100,000 humans. This finding varied from the systematic review results of Southeast Asia, which reported the incidence rates of rabies ranging between 0.1 and 117.2 per 100,000 persons (29). Geographical differences might be the reason for the variations.

rephrase the last sentence and make it the overarching statement. probably put it on the first line of the paragraph-385

Wondershare PDFelement

Among the animal studies included in the current review, the first and second highest records of incidence rates of rabies, 1880.19 (15) and 911.16 (19), were reported from cattle, while a relatively smaller incidence of rabies, 412.83 per 100,000, was reported from dogs (20). These different figures between cattle and dogs might indicate that a single rabid dog can infect many cattle during the episode of the disease. In general, diagnosis of rabies based on the clinical presentation alone might overestimate the recorded incidence rates. This possible justification could be supported by one of the included studies where higher estimates of incidence rates were recorded in clinically suspected animals than in laboratory-confirmed cases, 92.8 versus 70.2 in dogs and 5.7 versus 3.2 in cats (27).

To generate the pooled incidence rates for rabies and related outcome conditions, studies eligible for meta-analysis were segregated and utilized according to the outcomes reported. Twenty-three incidence data sets from nine studies were utilized for estimating the overall incidence rate of human exposure to suspected rabid animals. The annual pooled incidence rate of human exposure to suspected rabid animals per 100,000 humans was 33.65 (95% CI: 31.82 to 35.49) in the current meta-analysis. This result is much smaller than the pooled incidence rate of animal bites reported from Iran, 13.20 (95% CI: 12.10 to 14.30) per 1000 population (30). The differences might be due to geographical variation, under-reporting from Ethiopia, and/or differences in ways of defining rabies exposures. Higher incidence rates of canine rabies exposures were also reported from developed and developing regions of the world: Eastern Europe (120.9 from Croatia, 188.77 from Serbia, and 157.98 from Ukraine) and North Africa (277.15 from Algeria and 185.71 from Morocco) per 100,000 populations (31). The variation might be due to the difference in evidence generation methods; in our case, a systematic search was conducted, but in the other way, data with higher figures might be preferably presented.

and or differences in the definition of rabies exposure-401

402- are also reported

406-data with higher figures is not an evidence generation method.

The reliability of the meta-analysis result was demonstrated by the sensitivity analysis results for the incidence of human exposure to rabid animals, which were consistent with the overall effect size when most of the incidence data were eliminated one at a time. In a few cases, however, the overall results were noticeably changed when two datasets from the study reported by Mesfin (28) were omitted one

by one, giving the pooled incidence rates of 64.84 (95% CI: 60.97 to 68.70) and 63.61 (95% CI: 59.85 to 67.37) per 100,000 populations, indicating that these incidence data were extremely small compared with others. The Egger's regression test was performed to check for publication bias and obvious publication bias was detected among the publications that reported the incidence of human exposure to suspected rabid animals (Intercept = 22.06, 95% CI: 18.13 to 26.00; P<0.001). This result might show that the missing studies differ systematically from the observed studies.

It is important to note that in rabies-endemic countries, trustful reports of incidence rates of rabies and rabies exposure are lacking, especially in developing countries. So far, it has been described that official reports underestimated the true number of human rabies cases as well as their exposures (4, 32). In this manner, the Ethiopian national annual estimate from official reports indicated exposure cases of 12 per 100,000 populations (33). The current meta-analysis reported an annual pooled incidence rate of 33.65 (95% CI: 31.82 to 35.49) human exposures per 100,000 populations, which is almost three times the one previously officially reported.

In the current meta-analysis of human rabies exposures, considerable heterogeneity was recognized for the pooled estimate. From this, we could understand that variations existed in the effect sizes between the individual studies (Q (df) = 11222.628 (22) with *P*<0.001). The presence of heterogeneity among studies could also be explained by the heterogeneity statistics, I-squared (I^2), which indicated that about 99.804% of the variability in the effect-size estimates was due to the actual differences between studies. A further subgroup analysis was performed by regions to explore whether regions could be the source of heterogeneity between the individual studies included in determining the incidence of human exposure to rabid animals. Substantial heterogeneities were also seen from the subgroup analysis between regions (Q (df) = 2481.082 (3); P<0.001), indicating that regions of the studies would be sources of heterogeneity. Regionally, the pooled incidence rates of human exposure to suspected rabid animals ranged from 5.52 (95% CI: 3.26 to 7.79) to 138.91 (95% CI: 129.79 to 148.03) per 100,000 persons. The highest pooled incidence rate was recorded in Oromia, 138.91 (95% CI:

129.79 to 148.03), followed by Tigray, 83.32 (95% CI: 80.25 to 86.39), while the smallest estimate was from Amhara, 5.52 (95% CI: 3.26 to 7.79), followed by Addis Ababa, 7.58 (95% CI: 5.25 to 9.91). The reasons for the differences among the regions could be due to the following: individuals who are exposed to rabies often prefer to see traditional healers for the diagnosis and treatment of the disease because of their cultural background, lack of knowledge, or limited accessibility to medical treatment.

In determining the pooled incidence rate of suspected human rabies, six studies with 14 datasets were included, providing an effect size of 4.57 (95% CI: 2.93 to 6.21) per one million humans. This finding was significantly higher than the incidence of human rabies in China, 0.86 per 1,000,000 population (34). The difference might be due to the extensive implementation of surveillance systems to determine the real situation of rabies as well as the extensive dog vaccination in China (34), in contrast to the low coverage of dog vaccination (7, 11) and lack of active surveillance in Ethiopia. Our result was also higher than the report from sub-Saharan Africa with 1.4 cases (95% CI: 0.5–4.0) per 1,000,000 populations and lower than the one from South Asia with 9.2 cases (95% CI: 4.7–18.1) per 1,000,000 populations (34). Geographical variations might be the reason for the differences.

For the overall pooled incidence of suspected human rabies, sensitivity analysis was undertaken, and one study reported by Menghistu and others found an outlier (26). When the outlier was removed, the combined effect of the rest of the studies/datasets gave a very small pooled incidence, <1 per one million. However, the overall results were not noticeably changed by omitting each of the other studies/datasets. Among the publications included for determining the incidence of human rabies, it was shown that the missing studies differed systematically, as confirmed by Egger's regression test (Intercept = 4.01505, 95% CI: 1.43272 to 6.59738; P = 0.0027).

In the present review, five or fewer studies were included to determine the pooled incidence rates of suspected rabies in five different types of animals: 5 studies for cattle, 4 for dogs, 3 for equines, and 2 studies each for cats and goats. The highest pooled incidence was recorded in dogs, 120.99 (95% CI: 46.29 to 195.69), followed by equines, 19.57 (-1.85 to 40.98), and then cattle, with 18.08 (95% CI: 1 to

Wondershare PDFelement

35.15) per 100,000 populations. In Ethiopia, rabies is a potential problem for domestic animals such as cattle and others, as many households own dogs, usually for herding livestock or guarding property. This is because dogs are kept in close contact with cattle and other animals, transmitting the virus to them through a bite. By affecting cattle, rabies may have extensive economic impacts at the household and country levels, in addition to the effects on human health (35).

Regarding the associated factors of exposure to rabid animals, some reports included in the review described the relationship between certain socio-demographic features and the occurrence of rabies or its exposures. It was revealed that males were more exposed to rabid animals (21, 25). This could be due to the working environment of males, where they usually stay outside the home where they encounter stray dogs, or because male children have the habit of playing outside their homes with their age mates. Compared with adults, children <15 years old were more likely to be exposed to rabid animals (16, 17), and most human rabies deaths occurred among children (26). This might be explained by the fact that children have an interest in playing with pets, such as dogs and cats because they have poor awareness about rabies and its consequences. On the contrary, one study reported that adults were more affected (25). Contradictory reports were also reported regarding the residence and incidence of rabies cases. Two studies reported that rural inhabitants were more prone to exposure to rabid animals (16, 20). Another study reported that urban residents were more exposed (21). Such discrepancies require more explanations through further investigations.

A limited number of studies reported what measures were taken once humans were exposed to suspected rabid animals or the status of vaccination of dogs/animals inflicting bites. Almost 99% of individuals exposed to rabid animals had completed the required doses of nerve tissue vaccines (17). On the other hand, all the dogs inflicting unprovoked bites were unvaccinated (25). These reports indicate that the absence or low coverage of dogs' vaccination is one of the major problems that increases the risk of rabies and the costs associated with post-exposure treatments.

In the current systematic review and meta-analysis, some limitations were noted. Among them, one is that we found substantial heterogeneity in the estimation of the incidence rates of our outcome conditions across the included studies. In this regard, regions were identified as one of the sources of heterogeneity for the incidence of rabies exposures in humans. However, the other possible moderators for the heterogeneities have not been identified because of the difficulty of using such variables. The majority of the included studies were retrospective, where diagnosis bias could have occurred. The other problem was that the amount of data available per region was variable, even with no reports from the majority of the regions. Hence, the results from the review have to be cautiously interpreted.

Despite the above-highlighted limitations, this review and meta-analysis have some strengths. It used a predefined and registered protocol; this may ensure the reliability and scientific nature of the study. Many databases of medical literature were exhaustively searched for this review, which helped to retrieve a possible large number of studies available over long publication years. Furthermore, the search for published studies was supported by free online search engines. In all the phases of the review process, three independent investigators were also involved. In the original studies included in the review, sufficient data existed to answer our objectives.

Conclusions and Recommendations

The current study showed a high pooled incidence rate of human exposure to rabid animals. The overall incidence rates of rabies in humans and animals were also alarming. As rabies is one of the neglected zoonotic diseases, research in this area is limited in Ethiopia. The summarized report from our study provided important pictures of the risk of rabies, alarming that attention should be paid to the prevention and control of rabies in the country. As the high number of unvaccinated dogs is a major factor in the incidence of rabies and its associated public impacts, increasing the vaccination coverage of dogs is mandatory. This can be done through canine vaccination campaigns that can boost herd immunity and reduce the risk of human rabies exposure, necessitating strong governmental concerns and persistent community mobilization. Overall, integrating rabies prevention and control with other

Wondershare PDFelement

disease programs and strengthening intersectoral and transdisciplinary collaboration through one health approach is required.

Ethical approval: Not applicable.

Funding Statement: The authors received no funding for this work.

Competing interests: The authors have declared that no competing interests exist.

Data availability statement: All relevant data are within the paper and its supporting information files.

Supporting Information

Supplementary Information – I

Supplementary Information – II

Supplementary Information - III

Authorship contributions: Conceptualization of the work (GBH, AM, BTR); data extraction, article screening, and quality assessment (BTR, WT, DE); analysis (BTR); Interpretation (BTR, WT, DE, GBH, AM), development of the initial draft of the manuscript (BTR); critical revisions for the intellectual content of manuscript (BTR, GBH, AM); study supervision (GBH, AM). All authors reviewed and approved the final draft of the manuscript. The corresponding author (BTR) was responsible for the decision to submit the manuscript for publication.

References

1. WHO expert consultation on rabies: second report: World Health Organization; 2013.
2. WHO. Rabies fact sheets. 20 September 2023 [Available from: https://www.who.int/news-room/fact-sheets/detail/rabies (accessed October 05/2023).
3. Fekadu M, Endeshaw T, Alemu W, Bogale Y, Teshager T, Olson JG. Possible human-to-human transmission of rabies in Ethiopia. Ethiop Med J. 1996;34(2):123-7.
4. Hampson K, Coudeville L, Lembo T, Sambo M, Kieffer A, Attlan M, et al. Estimating the global burden of endemic canine rabies. PLoS Negl Trop Dis. 2015;9(4):e0003709.
5. Coetzer A, Kidane AH, Bekele M, Hundera AD, Pieracci EG, Shiferaw ML, et al. The SARE tool for rabies control: Current experience in Ethiopia. Antiviral Res. 2016;135:74-80.
6. Yimer E, Newayeselassie B, Teferra G, Mekonnen Y, Bogale Y, Zewde B, et al. Situation of rabies in Ethiopia: a retrospective study 1990-2000. Ethiopian Journal of Health Development. 2002;16(1):105-12.
7. CDC. Rabies in Ethiopia. September 28, 2017. [Available from: https://www.cdc.gov/worldrabiesday/ethiopia.html (accessed October 05, 2023).
8. Beyene TJ, Mourits MC, Kidane AH, Hogeveen H. Estimating the burden of rabies in Ethiopia by tracing dog bite victims. PLoS One. 2018;13(2):e0192313.

Wondershare PDFelement

9. Abela-Ridder B. Rabies: 100 per cent fatal, 100 per cent preventable. J Vet Rec. 2015;177(6):148-9.

10. Ali A, Mengistu F, Hussen K, Getahun G, Deressa A, Yimer E, et al. Overview of rabies in and around Addis Ababa, in animals examined in EHNRI zoonoses laboratory between, 2003 and 2009. Ethiopian Veterinary Journal. 2010;14(2):91-101.

11. Abdella S, Ahmed K, Salim B, Ashenefe B, Mulugeta Y, Girma E, et al. High rabies burden and low vaccination status among dogs inflicting bite in Addis Ababa: an urgent call for action. The Journal of Infection in Developing Countries. 2022;16(08.1):15S-9S.

12. Belete S, Meseret M, Dejene H, Assefa A. Prevalence of dog-mediated rabies in Ethiopia: a systematic review and Meta-analysis from 2010 to 2020. One Health Outlook. 2021;3(1):16.

13. Page MJ, McKenzie JE, Bossuyt PM, Boutron I, Hoffmann TC, Mulrow CD, et al. The PRISMA 2020 statement: an updated guideline for reporting systematic reviews. Systematic reviews. 2021;10(1):1-11.

14. Beyene TJ, Mourits MCM, Kidane AH, Hogeveen H. Estimating the burden of rabies in Ethiopia by tracing dog bite victims. PLoS One. 2018;13(2).

15. Jibat T, Mourits MC, Hogeveen H. Incidence and economic impact of rabies in the cattle population of Ethiopia. Prev Vet Med. 2016;130:67-76.

16. Kabeta T, Tigre W, Deressa B. Occurrence of suspected rabies cases in humans Jimma zone and surrounding areas, south West Ethiopia. International Journal of Basic Applied Virology. 2014;3(2):28-34.

17. Ramos JM, Melendez N, Reyes F, Gudiso G, Biru D, Fano G, et al. Epidemiology of animal bites and other potential rabies exposures and anti-rabies vaccine utilization in a rural area in Southern Ethiopia. Ann Agric Environ Med. 2015;22(1):76-9.

18. Wakgari M, Getachew G, Van't Klooster G, Tewolde N, Kivaria F, Bebay C. Participatory epidemiological study on the burden of rabies in animals and humans in three districts of Buno Bedele Zone, West Ethiopia. Ethiopian Veterinary Journal. 2022;26(2):93-106.

19. Alemu YF, Jemberu WT, Mekuriaw Z, Abdi RD. Incidence and Predictors of Calf Morbidity and Mortality From Birth to 6-Months of Age in Dairy Farms of Northwestern Ethiopia. Frontiers in Veterinary Science. 2022;9.

20. Jemberu WT, Molla W, Almaw G, Alemu S. Incidence of Rabies in Humans and Domestic Animals and People's Awareness in North Gondar Zone, Ethiopia. PLoS Negl Trop Dis. 2013;7(5).

21. Yibrah M, Damtie D. Incidence of human rabies exposure and associated factors at the Gondar Health Center, Ethiopia: A three-year retrospective study. Infectious Diseases of Poverty. 2015;4(1).

22. Yizengaw E, Getahun T, Mulu W, Ashagrie M, Abdela I, Geta M. Incidence of human rabies virus exposure in northwestern Amhara, Ethiopia. BMC Infect Dis. 2018;18(1).

23. Ebuy Y, Alemayehu T, Reda M, Berhe M, Bsrat A. Community knowledge, attitude and practice on rabies, incidence in humans and animals and risk factors to rabies in selected districts of Tigray Region, Ethiopia. Nigerian Veterinary Journal. 2019;40(2):147-63.

24. Gebru G, Romha G, Asefa A, Hadush H, Biedemariam M. Risk factors and spatio-temporal patterns of human rabies exposure in northwestern tigray, ethiopia. Annals of Global Health. 2019;85(1).

25. Teklu GG, Hailu TG, Eshetu GR. High Incidence of Human Rabies Exposure in Northwestern Tigray, Ethiopia: A Four-Year Retrospective Study. PLoS Negl Trop Dis. 2017;11(1).

26. Menghistu HT, Hailu KT, Shumye NA, Redda YT. Mapping the epidemiological distribution and incidence of major zoonotic diseases in South Tigray, North Wollo and Ab'ala (Afar), Ethiopia. PLoS One. 2018;13(12).

27. Kidane A, Sefir D, Bejiga T, Deressa A, Pal M. Rabies in animals with emphasis on dog and cat in Ethiopia. Worlds Veterinary Journal. 2016;6(1):123.

28. Mesfin Z. A Retrospective Study of Suspected Human Rabies Exposure Cases in Addis Ababa, Ethiopia. American Journal of Health Research. 2022;10(3):44-50.

29. Ling MYJ, Halim AFNA, Ahmad D, Ramly N, Hassan MR, Rahim SSSA, et al. Rabies in Southeast Asia: a systematic review of its incidence, risk factors and mortality. BMJ open. 2023;13(5):e066587.

30. Abedi M, Doosti-Irani A, Jahanbakhsh F, Sahebkar A. Epidemiology of animal bite in Iran during a 20-year period (1993–2013): a meta-analysis. Tropical medicine health. 2019;47:1-13.

31. Taylor E, Vilas VDR, Scott T, Coetzer A, Prada JM, Alireza G, et al. Rabies in the Middle East, Eastern Europe, Central Asia and North Africa: Building evidence and delivering a regional approach to rabies elimination. Journal of infection and public health. 2021;14(6):787-94.

32. Taylor L, Knopf L, Prevention PfR. Surveillance of human rabies by national authorities–a global survey. Zoonoses Public Health. 2015;62(7):543-52.

33. Deressa A, Sefir D, Mamo H, Ali A, Akililu M, Getahun G, editors. Rabies diagnosis and surveillance for the period 2010–2012 in Ethiopia. 11th SEARG meeting, Dar es Salaam; 2013.

34. Ali M, Chang B, Isabel S, Morris SK, editors. Global epidemiology of Human Rabies: Systematic Review and Meta-Analysis. Open Forum Infectious Diseases; 2015: Infectious Diseases Society of America.

35. Okell C, Pinchbeck G, Stringer A, Tefera G, Christley R. A community-based participatory study investigating the epidemiology and effects of rabies to livestock owners in rural Ethiopia. Prev Vet Med. 2013;108(1):1-9.

Click here to access/download
Supporting Information
Supplementary Information - I (Search terms and strings).docx

Wondershare PDFelement

Click here to access/download
Supporting Information
Supplementary Information-II (Data extraction sheet).xlsx

Wondershare PDFelement

Click here to access/download
Supporting Information
Supplementary Information - III (Forest plots).pdf
