# Supplementary material for: Incidence, risk factors, and control of Rabies in Ethiopia: A systematic review and meta-analysis
Source: PLoS Negl Trop Dis. 2025 Mar 19;19(3):e0012874. doi: 10.1371/journal.pntd.0012874 (PMC11922250; doi:10.1371/journal.pntd.0012874)
Supplement: Attachment — Submitted filename: Response to Reviewers.pdf [file pntd.0012874.s007.pdf]

Date: December 21, 2024

Rebuttal Letter

Dear Reviewers and Editors,

First of all, we are grateful to the reviewers and editors for investing their time in evaluating our paper, and for providing valuable comments that surely improve the quality of our work.

Saying this, we would like to give responses/clarifications for the issues raised from the reviewers and editors of PLOS Neglected Tropical Diseases Journal as follows.

Reviewer concerns/comments: “While the authors have made some efforts to address the concern raised, they failed to justify why this investigation was conducted, including how it might support the country's rabies prevention and control efforts.”

Responses/clarifications:

We conducted this systematic review and meta-analysis because, to the best of our knowledge, no systematic review and meta-analysis was done in Ethiopia on the incidence rates of rabies and its exposure on humans as well as on animals. To fill such gaps, we are interested to inform the programmers and policy makers by performing the systematic review and meta-analysis on the incidence of rabies in human and different animals in Ethiopia.

Reviewer concerns/comments: “Clearly, Several studies have been done in Ethiopia that have quantified risk and provided helpful information (https://onehealthoutlook.biomedcentral.com/articles/10.1186/s42522-021-00046-7). Including the ones the authors reported. According to the CDC, rabies results in the death of 2,700 persons annually in Ethiopia, making it one of the highest death rates in the world (7). Furthermore, extrapolation of the district data to the national level indicated an annual estimate of approximately 3,000 human deaths, 194,000 DALYs, and 97,000 exposed persons, requiring on average 2 million USD in treatment costs per year countrywide (8).”

Responses/clarifications:

The systematic review and meta-analysis report of the study cited with ‘https://onehealthoutlook.biomedcentral.com/articles/10.1186/s42522-021-00046-7’ was

done on the prevalence of dog-mediated rabies in Ethiopia. Acknowledging the authors by citing (reference 12) this article, we used the result of this study to show the overall prevalence of rabies in humans and animals in Ethiopia. This is different from our systematic review and meta-analysis in that ours has focused on the pooled incidence rates. In epidemiology, prevalence and incidence are used for different purposes and to answer different research questions. "Prevalence" refers to the proportion of a population that has an event of interest at a specific point in time, while "incidence rate" measures the number of new cases of a disease that develop within a population over a specific period of time; essentially, prevalence captures the current burden of a disease, while incidence reflects the rate at which new cases are occurring. In our meta-analysis, we determined the pooled incidence rate of human rabies exposure, human rabies, and incidence rates of rabies in different animals.

The other studies that have been performed in Ethiopia are individual studies or reports that we included in the introduction section to show the situation of rabies. The other individual studies reporting incidence rates have been included in our systematic review and meta-analysis as per the inclusion and exclusion criteria set for our research.

Reviewer concerns/comments: “The authors' justification for conducting this study is not clear (119- 121) and cannot in any way support the planning of mass dog vaccination, as nowhere in the paper did the authors report maps of locations needing targeted intervention. Lines 119 to 121. However, the overall risk of rabies in a population has not yet been estimated in the country. Information on the rabies incidence rate is important for notifying the population at risk of the disease and for planning effective prevention, control, and elimination strategies.”

Responses/clarifications:

Unfortunately, the justification for conducting our study was not clear in our original manuscript. We would like to apologize for the misunderstanding and have now revised the paper to explain this better.” Here is the way we rephrased: … Information on the overall rabies incidence rates is important for notifying the population at risk of the disease and for planning effective prevention, control, and elimination strategies. However, to the best of our knowledge, the pooled incidence rates of rabies in human and animal populations have not yet been estimated in the country. The absence of such summarized data on the incidence of rabies, both in humans and animals, might be

among the major problems that cause negligent commitment to the control of rabies, as still Ethiopia is in the early stages of rabies control…

Regarding the issue of mass dog vaccination, we tried to show the gaps in the introduction section that vaccination coverage in Ethiopia is far below the optimal level (below 70%) to prevent canine rabies transmission in the country. For example, evidence indicated that only 27% of the dogs are vaccinated in Addis Ababa, the capital city of the country. This existing evidence showed that there is a wider difference between the recommended optimal dog vaccination coverage and the achieved vaccination coverage. Our current study also showed a significantly high pooled incidence rate of human exposure to rabid animals. Again, the overall incidence rates of rabies in humans and animals are also alarming. Hence, the findings from our study strongly support the planning of mass dog vaccination as 99% of human rabies exposure and rabies cases are associated with dog bites.

Regarding the 'maps of locations needing targeted intervention', w*e* thank the reviewer for this valuable suggestion and agree that it would be interesting to be addressed. However, it is beyond the objectives of our research.

Reviewer concerns/comments: "The manuscript is too lengthy and contains a lot of repetition (Lines 95 and 106 say the same thing) and contractions. For example, according to the authors, there is limited capacity for testing (Line 92); when you go further into Line 107; the authors reported 70.3% lab confirmation. The authors of this paper need to justify why this study was conducted by articulating findings from previous studies in the country and how their work will advance rabies prevention and control efforts in the country."

Responses/clarifications:

We accept the comment that the manuscript is too lengthy and it contained repetitions. We tried to reduce texts from the discussion section because these ideas are redundant as the reviewers mentioned. However, we have got difficulty in further summarizing of the manuscript due the fact that we have many outcome variables, such as incidence rate of human exposure to suspected rabid animals, incidence rate of rabies in humans, incidence rate of rabies in different animals as well as the associated factors of human rabies, its exposures, and control measures. As of the capacity for rabies testing concerned, we would like to explain that there is one national reference laboratory for confirming of rabies in the

country. In line with this, the 70.3% laboratory confirmed cases were a report from a single study done at the national reference laboratory. However, diagnostic laboratories for confirming rabies are lacking at the peripheries, or different corners of Ethiopia.

Finally, we would like to thank the reviewers and editors for evaluating our manuscript. We have tried to address all the reviewers' and editors' concerns in a proper way and believe that our paper has improved considerably. We would be happy to make further corrections if necessary and look forward to hearing from you soon.

Best regards!

Belay Tafa Regassa (Corresponding Author)
