# Supplementary material for: Incidence, risk factors, and control of Rabies in Ethiopia: A systematic review and meta-analysis
Source: PLoS Negl Trop Dis. 2025 Mar 19;19(3):e0012874. doi: 10.1371/journal.pntd.0012874 (PMC11922250; doi:10.1371/journal.pntd.0012874)
Supplement: Attachment — Submitted filename: Point-by-point responses to reviewers comments.docx [file pntd.0012874.s006.docx]

**Point-by-point Responses to Reviewer’s comments/concerns**

**Manuscript: Incidence, risk factors, and control of Rabies in Ethiopia: A systematic review and meta-analysis**

| **S/N** | **Manuscript section** | **Key Review Criteria Required for Acceptance?** | **Reviewer’s responses/comments/or concerns** | **Point-by-point authors’ corrections, responses, or explanations** |
| --- | --- | --- | --- | --- |
| 1 | Methods | - Are the objectives of the study clearly articulated with a clear testable hypothesis stated? - Is the study design appropriate to address the stated objectives - Is the population clearly described and appropriate for the hypothesis being tested? - Is the sample size sufficient to ensure adequate power to address the hypothesis being tested? - Were correct statistical analysis used to support conclusions? - Are there concerns about ethical or regulatory requirements being met? | Reviewer #1: no response | - |
|  |  |  | Reviewer #2:   - the objectives of the study were clearly articulated and clear - Yes, the study design, systematic review is appropriate and rigorously followed to address the objective - The population was clearly described and suitable for testing - The selected articles used for the study was based on the selection criteria, which for this study was clearly selected and was significant to support the results produced. - **Yes, the statistical analysis used was correct even though poorly presented and it will be important for the author to present in a simplified manner.** - No concerns on ethics or regulatory requirement as the authors registered the selected articles in PROSPERO with number CRD4202346879 | - We modified the data presentations of the statistical outputs based on the reviewers’ comments. We have removed some of the parameters, such as relative weight, z-values and their p-values from figures 2, 5, and 6, which were not directly used for result interpretations. |
| 2 | Results | - Does the analysis presented match the analysis plan? - Are the results clearly and completely presented? - Are the figures (Tables, Images) of sufficient quality for clarity? | Reviewer #1: (No Response) | - In most of the papers included in the SR and MA, a single published article in a given publication year has a number of datasets of incidence rates due to the differences in person-years. In this aspect, we couldn’t analyze the trends of incidence rates by publication year. We accept this as a weakness, and it would be better to acknowledge as a limitation. - In general, a limited number of studies reported the risk factors for human rabies exposures or deaths. According to the reviewers’ comments, we have separated into separate paragraphs. In addition to age and sex, we also explored the residence as one of the factors. - We have addressed the issue of figures by harmonizing the data presentations. We omitted some of the parameters which were not directly implicated in result interpretations, as mentioned above (under S/N. 1, method’s section). |
|  |  |  | Reviewer #2   - Yes it does, the analysis was able to put data from different sources and different years and calculate the incidence rate. **The weakness however, is that the differences in the years might affect the results produced such that the current rate might not be real due to the cumulative approach.** However, it responded to the research question and is still informative. - **The results for risk factors were not clearly presented; however, the paragraph is loaded with lots of information which could be clearly presented. For example, Sex and age are associated factors. They should be presented and discussed in different paragraphs. Also, the author could explore some other factors associated to rabies exposure to improve on the qualitative data.** - **The figures and tables created ok except for those of incidence rates which are different and should be harmonized.** |  |
| 3 | Conclusions | - Are the conclusions supported by the data presented? - Are the limitations of analysis clearly described? - Do the authors discuss how these data can be helpful to advance our understanding of the topic under study? - Is public health relevance addressed? | Reviewer #1: (No Response) | - As per the reviewers’ comments, we have tried to specify to whom the recommendations are concerned. Because of the scarcity of information on rabies incidence and its associated factors, we have also recommended further individual studies from various corners of the country. - Please, note that we have included the limitations and strengths of the study under the discussion section; would you look at the last two paragraphs of the discussion section. |
|  |  |  | Reviewer #2   - The conclusion is supported by data **but could be improved upon by including some more information as per the objective and expand a little on the recommendation and possibly highlighting to whom the study is addressed to or services concerned.** - **The limitations are not defined and should be included in the study.** - Yes, they showed evidence of high incidences and showed different rabid animals. **However, the authors should mention the opportunities for research in this domain based on this study.** - Public health relevance is not directly addressed. The authors addressed health in general. |  |
| 4 | Editorial and Data Presentation Modifications? | Use this section for editorial suggestions as well as relatively minor modifications of existing data that would enhance clarity. If the only modifications needed are minor and/or editorial, you may wish to recommend “Minor Revision” or “Accept”. | Reviewer #1: (No Response)  Reviewer #2:   - The article needs minor revisions, such as grammatical errors, harmonization of the tables. - **Also, the author should describe the data in terms of years.** For example, how many articles were collected in 2013 and how many in 2011? This will help the reader not only understand possible trends, but will give some reliability to the data. - Also, **the authors should state the limitation of the data analysis. One of them being that, it is not stated the source of the data; some of the studies might be using same data etc.** All of that is not very clear in the study. - For the qualitative data, some description of the method used should be mentioned and the presentation improved upon. The author could also ensure that just **sex and age are the only human exposure factor to rabies.** Apart from that minor revision | As explained in the above sections, we have harmonized the tables. E.g. Figures 2, 5, and 6 were modified.  According to comments, the number of articles collected in each publication year has been described in Table 1.  In the limitation, we have acknowledged that trends analysis by year of publication was not possible due to the different datasets in most of the publication years.  We have also explained that residence was one of the associated factors. |
| 5 | Summary and General Comments | Use this section to provide overall comments, discuss strengths/weaknesses of the study, novelty, significance, general execution and scholarship. You may also include additional comments for the author, including concerns about dual publication, research ethics, or publication ethics. If requesting major revision, please articulate the new experiments that are needed. | Reviewer #1:  Regassa et al. conducted a systematic review and meta-analysis on the incidence, risk factors, and control of rabies in Ethiopia. They reported a high pooled incidence rate of human exposure to rabid animals and significant overall incidence rates of rabies in both humans and animals. The authors emphasized the need for strengthening intersectoral and transdisciplinary collaborations using a One Health approach to enhance rabies prevention and control efforts. However, the introduction of the study is currently too lengthy. To improve the introduction, the authors should consider providing a concise background.  The discussion section is lengthy and contains several repetitions of the results. Previous studies conducted in Ethiopia and elsewhere have emphasized many of the discussion points. Therefore, the authors should focus on synthesizing key findings and providing new insights or interpretations. They should avoid repetition of results and instead highlight novel contributions or implications of their study. Additionally, the authors could compare their findings with previous studies from Ethiopia and other regions to underscore similarities, differences, or advancements in knowledge. | Accepting the reviewers’ comments, we reduced the ‘Introduction section’.  Obviously, we have a number of outcomes of interest: human rabies exposures, human rabies deaths, rabies in various domestic animals, and factors associated with rabies as well as rabid animals’ exposures. This made our discussion section lengthy to some extent. We removed some paragraphs introducing the section. |
|  |  |  | Reviewer #2:   - Strength   - very important but neglected diseases   - used both qualitative and quantitative analysis   - clear inclusion and exclusion criteria opportunity for more research on rabies across SSA - Weakness-   - No year limit for articles   - Comparison of results with other countries in the discussion is not very visible. - The study is not completely novel however, opens widows for rigorous data collection on rabies and its associated risk factors. Generally, the study is very interesting and fairly exploited especially at the level of the qualitative study. | The year limit for the articles has been mentioned under the ‘Methods section’ sub-section ‘Inclusion and exclusion criteria’ as follows: … studies conducted in Ethiopia until October 6, 2023, from the time of the inception of each database …  Regarding the comparison of results from other countries, we tried our best to search for published Systematic reviews and meta-analysis in line with our outcomes of interest. Unfortunately, such studies are scarce. |
| 6 | **Line by line comments on the built manuscript’s pdf** | | **Line -35:** Rephrase considering plural: (A published article search was……) | Corrected as ‘Published articles search was… |
|  |  |  | **Line -93:** ‘….contribute’ should be corrected as ‘…..contributes…  The paragraph should discuss dog management as stated in the first line. | The first statement was merged into paragraph-2. |
|  |  |  | **Line -197:** state the reasons for exclusion  **Line -198:** state the scoring category and the criteria | The reasons for exclusion were already stated in the PRISMA 2020 flow diagram (Figure 1).  The issues of quality assessment, such as tools used and scoring system, have been described under the ‘Methods’ section (sub-section, **Study selection and quality assessment**) |
|  |  |  | Under subtopic **‘Characteristics of the included studies’ (Line -202),** describe the articles by year of publication. | As per the comments, we indicated the year of publication of articles in Table 1. |
|  |  |  | Under subtopic **‘Risk factors of exposure to rabid animals or rabies in humans, and control measures’ (Line -273),** discuss each risk factor separately. e.g. - Age ....sex... etc | We accepted the comments and discussed separately. |
|  |  |  | **Line -401:** correct as ….and/or differences in the definition of rabies exposure | Corrected. |
|  |  |  | **Line -402:** correct as **…**are also reported. | Corrected. |
|  |  |  | **Line 406: data with higher figures is not an evidence generation method** | The statement has been modified. |
